# Structure of the Wnt–Frizzled–LRP6 initiation complex reveals the basis for coreceptor discrimination

**DOI:** 10.1073/pnas.2218238120

**Published:** 2023-03-09

**Authors:** Naotaka Tsutsumi, Sunhee Hwang, Deepa Waghray, Simon Hansen, Kevin M. Jude, Nan Wang, Yi Miao, Caleb R. Glassman, Nathanael A. Caveney, Claudia Y. Janda, Rami N. Hannoush, K. Christopher Garcia

**Affiliations:** ^a^HHMI, Stanford University School of Medicine, Stanford, CA 94305; ^b^Department of Molecular and Cellular Physiology, Stanford University School of Medicine, Stanford, CA 94305; ^c^Department of Structural Biology, Stanford University School of Medicine, Stanford, CA 94305; ^d^Graduate School of Medicine, Dentistry and Pharmaceutical Sciences, Okayama University, Okayama 700-8530, Japan; ^e^Department of Early Discovery Biochemistry, Genentech, South San Francisco, CA 94080; ^f^Princess Máxima Center for Pediatric Oncology, 3584 CS Utrecht, Netherlands

**Keywords:** Wnt signaling, cryo-EM, protein engineering, structural biology, crystallography

## Abstract

Wnt morphogens are critical for embryonic development and tissue regeneration. Canonical Wnts form ternary receptor complexes composed of tissue-specific Frizzled (Fzd) receptors together with the shared LRP5/6 coreceptors to initiate β-catenin signaling. The cryo-EM structure of a ternary initiation complex of an affinity-matured XWnt8–Frizzled8–LRP6 complex elucidates the basis of coreceptor discrimination by canonical Wnts by means of their N termini and linker domains that engage the LRP6 E1E2 domain funnels. Chimeric Wnts bearing modular linker “grafts” were able to transfer LRP6 domain specificity between different Wnts and enable non-canonical Wnt5a to signal through the canonical pathway. Synthetic peptides comprising the linker domain serve as Wnt-specific antagonists. The structure of the ternary complex provides a topological blueprint for the orientation and proximity of Frizzled and LRP6 within the Wnt cell surface signalosome.

## Significance

The structure of the signaling-competent Wnt–Frizzled–LRP6 complex reveals the basis for initiating canonical Wnt/β-catenin signaling. An unusual “tandem anchoring” mode for Wnt binding to LRP6 via two flexible loops of Wnt clarifies the natural topology of the canonical Wnt signaling complex that could serve as a structural blueprint for designing “surrogate” Wnts to mimic the endogenous Wnt-induced dimerization mode. Grafting these loops onto different Wnts changes their binding selectivity for LRP6 extracellular domains. The modular nature of Wnt discrimination of LRP6 from the “non-canonical” coreceptors opens the possibility of swapping the linker domain to create “designer Wnts” with altered functions in regenerative medicine and cancer biology.

Wnt morphogens play essential roles in embryonic development and tissue regeneration by orchestrating stem cell proliferation and differentiation (1–4). To initiate signaling, Wnt simultaneously engages the extracellular cysteine-rich domain (CRD) of a seven-transmembrane receptor Frizzled (Fzd) and an extracellular domain (ECD) of the coreceptors LRP5 or LRP6. The activated Wnt–Fzd–LRP5/6 complex inhibits the destruction complex, leading to the accumulation of the transcriptional coactivator β-catenin which translocates to the nucleus and induces the expression of Wnt target genes (5).

Due to its crucial function in cell fate decisions, aberrant Wnt activation is linked to various diseases, including multiple types of cancers, making it an attractive therapeutic target by Wnt signaling antagonists (6). On the contrary, Wnt agonism can be harnessed for regenerative medicine upon tissue injury or in bone and hair losses (7). Engineered bispecific dimerizers that induce Fzd–LRP6 proximity can act as surrogate agonists in order to therapeutically exploit Wnt’s powerful functions in stem cell expansion and tissue renewal (8–11). These molecules, as well as Norrin (12, 13), demonstrate that induced proximity between Fzd and LRP5/6 is the principal mechanism of signal activation. However, the signaling strength of such agonists appears highly dependent on the relative orientation and proximity of the Fzd–LRP6 heterodimer.

The previous Wnt–Fzd crystal structures revealed the binding mode of canonical Wnts, *Xenopus* Wnt8 (XWnt8) and Wnt3, to their primary receptor Fzd (14, 15). However, these binary complexes lacked LRP5/6 that interact with Wnt through their ECD consisting of four Tyr-Trp-Thr-Asp (YWTD) β-propellers flanked by epidermal growth factor (EGF)-like domains. It has been shown that Wnts can be clustered into different families based on their binding preferences to distinct LRP6 domains (16, 17). It remains to be determined how different Wnts can bind to different regions in LRP6 in contrast to the fact that Wnts bind to the Fzd CRD (Fzd_CRD_) in a very conserved manner. Furthermore, it is also unclear how non-canonical Wnts have structurally evolved to bypass LRP5/6 coreceptor requirements for their function. For these reasons, structural information on a Wnt–Fzd–LRP6 ternary signaling complex is important for understanding the geometric requirements (i.e., orientation and proximity) of the receptors for signaling initiation, how Wnts differentially bind to LRP6, and finally, for rationally optimizing surrogate Wnts to achieve the natural topology of the endogenous Wnt signaling complex.

## Results

### Wnt Engineering.

**Fig. 1.**
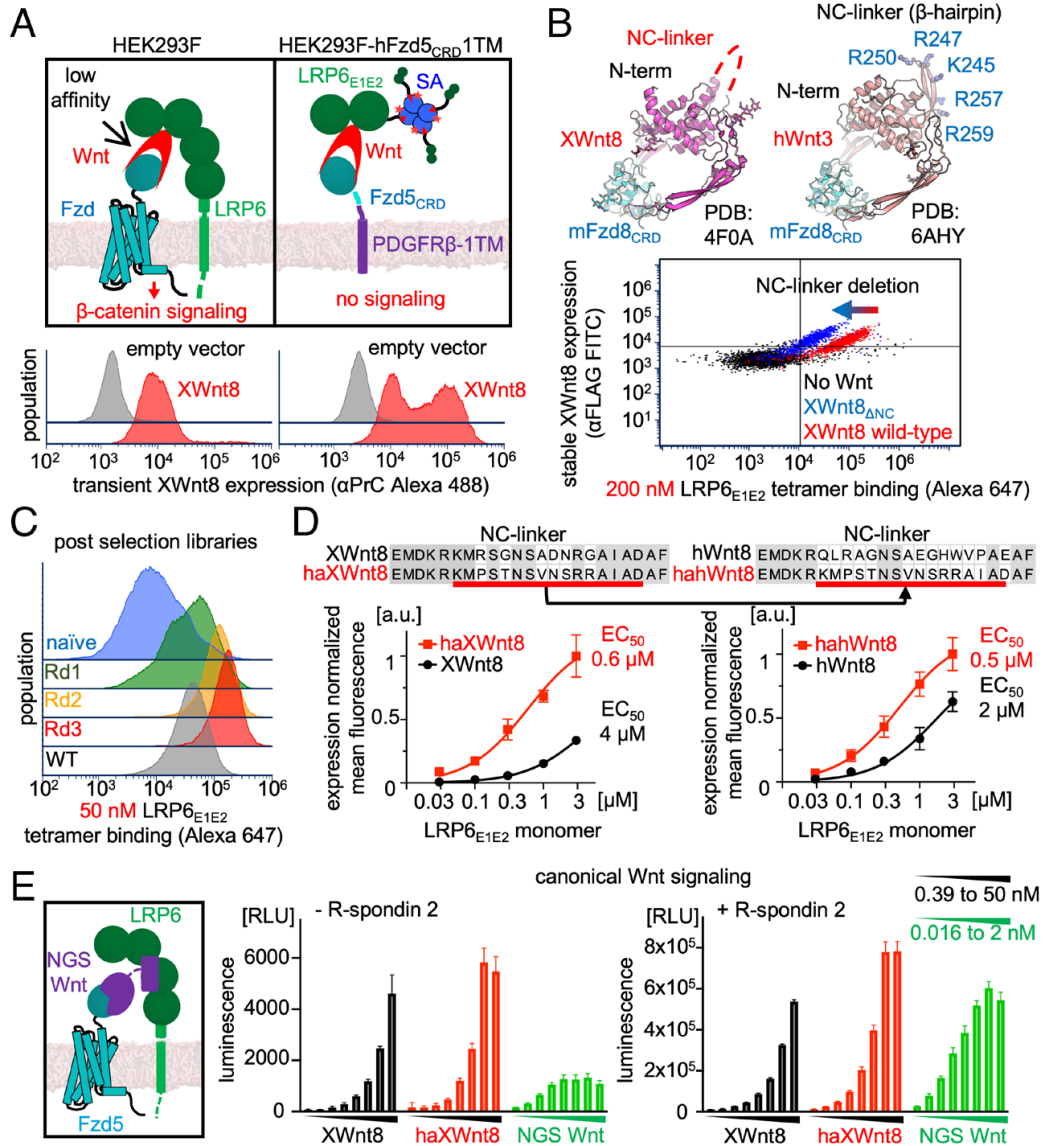
Engineering and characterization of haXWnt8. (*A*) Schematic for the cell-surface Wnt display and signaling of wild-type HEK293F and HEK293 expressing hFzd5_CRD_-1TM (*Top*). Wnt expression detected by protein C (PrC) epitope tag on Wnt (*Bottom*). (*B*) Ribbon models of XWnt8–mFzd8_CRD_ (PDB 4F0A) and hWnt3–mFzd8_CRD_ (PDB 6AHY) (*Top*). The NC-linker is missing in the XWnt8 crystal structure, likely reflecting its flexibility. Cell surface expression and LRP6_E1E2_ tetramer binding on hFzd5_CRD_-1TM cell line in the absence or presence of wild-type XWnt8 or XWnt8_ΔNC_ (*Bottom*). (*C*) Improvements in LRP6_E1E2_ tetramer binding to the XWnt8 libraries during selection compared to the wild-type XWnt8 single clone. Data were analyzed with FCS Express 7. (*D*) LRP6_E1E2_ monomer binding to the haXWnt8 and wild-type XWnt8 (*Left*) and hahWnt8 and wild-type hWnt8 (*Right*). The squares and dots indicate the means of experimental values, and the error bars represent SD. Data were analyzed with Prism 9. The NC-linker sequences are presented on top of the binding curves. (*E*) Top-flash signaling assays with recombinant wild-type XWnt8, haXWnt8, and next-generation surrogate (NGS) Wnt (10) (*Left*) in the absence or presence of R-spondin 2. With twofold serial dilution, the agonist concentrations were 0.39 nM to 50 nM for XWnt8s and 0.016 nM to 2 nM for NGS Wnt. The bar graphs show the means of experimental values, and the error bars represent SD. Data were analyzed with Prism 9. Dose–response experiments were performed in triplicate, and representative data were shown from two independent experiments.

To reconstitute a stable Wnt–Fzd–LRP6 complex for cryo-electron microscopy (cryo-EM) analysis, we first sought to engineer a Wnt that binds with increased affinity to LRP6. However, since Wnt proteins are lipidated and insoluble in the absence of detergents (18), they are problematic for conventional cell surface display methods commonly used for soluble proteins (8). We devised a strategy to display a library of *Xenopus* Wnt8 (XWnt8) variants bound to human Fzd5_CRD_ (hFzd5_CRD_) on the cell surface of mammalian cells and select XWnt8 variants that bind with higher affinity to the ECD of LRP6 (Fig. 1 *A*, *Top*). In order to prevent Wnt-induced downstream signaling and internalization (Fig. 1 *A*, *Top* *Left*), we replaced the transmembrane region of hFzd5 with a single transmembrane helix (19) (Fig. 1 *A*, *Top* *Right*), which improved the cell surface display of XWnt8 (Fig. 1 *A*, *Bottom*). The cell surface XWnt8–hFzd5_CRD_ complex bound human LRP6 E1E2 (hLRP6_E1E2_) (17, 20), which was tetramerized to increase the affinity through avidity effect for detecting by flow cytometry (Fig. 1*B*). Deleting the NC-linker of XWnt8 (XWnt8_ΔNC_), which has previously been reported to mediate LRP6 binding in the context of Wnt3 signaling (15, 21), substantially decreased, but did not completely abolish, hLRP6_E1E2_ binding (Fig. 1*B*). These indicated that the wild-type XWnt8–Fzd5_CRD_ complex displayed on the cell surface is functional regarding LRP6 recognition, and that the NC-linker is a primary LRP6 binding site, but additional interactions beyond the NC-linker may contribute to LRP6 binding. We then created “soft-randomized” libraries of XWnt8 variants that harbored mutations in the NC-linker (residues 222 to 234) with an experimental diversity of approximately 1 million unique sequences (*SI Appendix*, Fig. S1*A*). The libraries were designed to introduce sparse mutations within the NC-linker that would exhibit enhanced binding energetics to LRP6 while maintaining the overall structural integrity of the natural linker.

After three rounds of fluorescence-activated cell sorting (FACS) selection for hLRP6_E1E2_ binding (Fig. 1*C* and *SI Appendix*, Fig. S1*B*), we isolated five XWnt8 clones with improved hLRP6_E1E2_ tetramer binding relative to wild-type XWnt8 (*SI Appendix*, Fig. S2*A*). We tested the XWnt8 variant with the highest expression (high-affinity XWnt8, haXWnt8) for hLRP6_E1E2_ monomer binding on cells, which had acquired six amino acid substitutions (Fig. 1*D*). The haXWnt8 variant had at least sixfold improved binding affinity (EC_50_) relative to wild-type XWnt8 (Fig. 1 *D*, *Left*) and grafting this linker into the corresponding region of human Wnt8 (hWnt8) yielded a similarly higher affinity (Fig. 1 *D*, *Right* and *SI Appendix*, Fig. S2*B*). We performed super TOPflash Wnt reporter assays using recombinant XWnt8 or haXWnt8 in comparison with surrogate (NGS) Wnt (10) (Fig. 1*E*). haXWnt8 showed more potent β-catenin signaling than the wild-type XWnt8 both in the absence and presence of R-spondin 2. In addition, we noticed that the surrogate Wnt showed a substantially better EC_50_ than the Wnts due to its high affinity but with a lower maximum effect (E_max_).

### Reconstitution of the Soluble Wnt Ternary Complex and Structural Analysis.

**Fig. 2.**
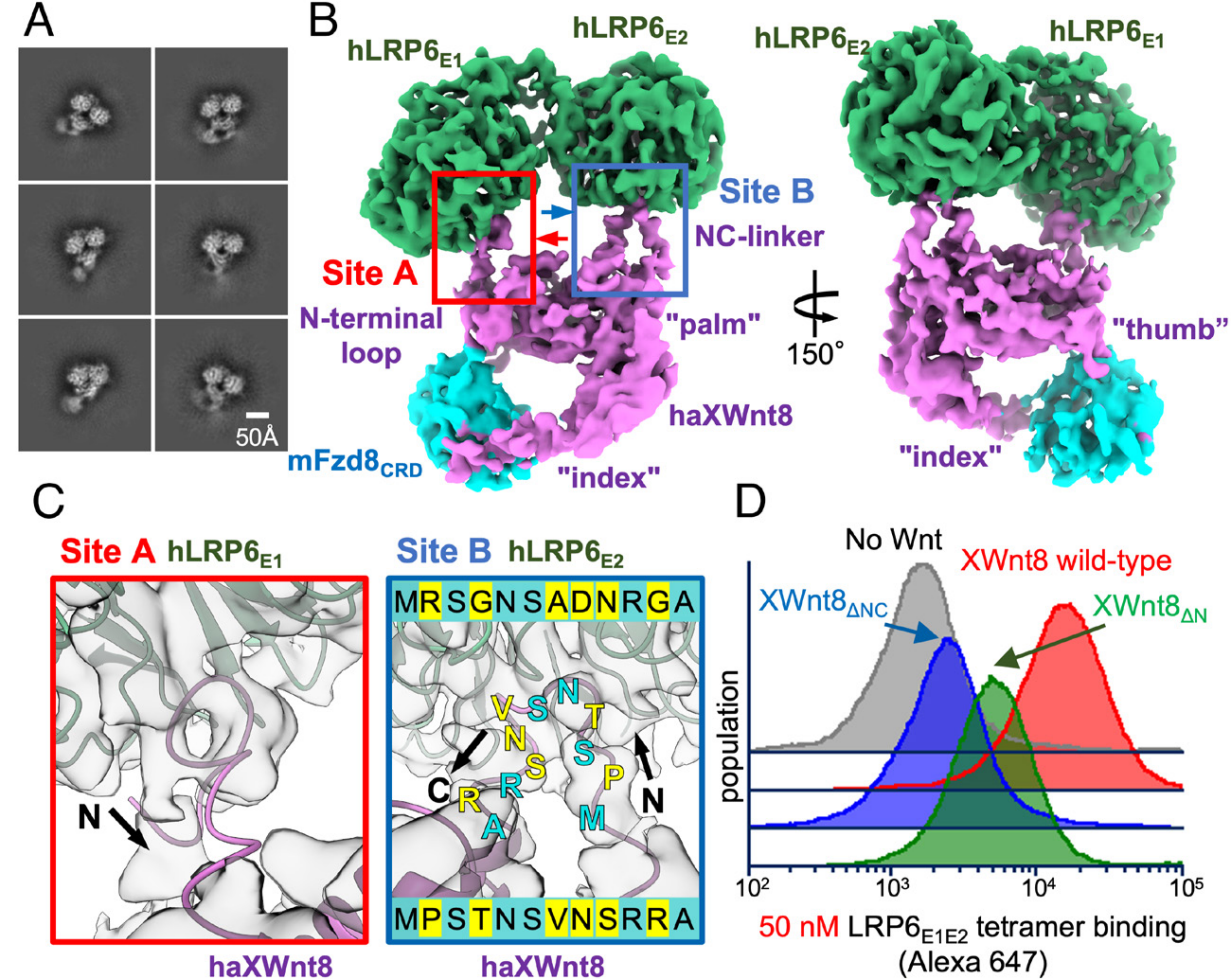
Cryo-EM analysis of haXWnt8–mFzd8_CRD_–hLRP6_E1E2_. (*A*) Representative 2D class averages of haXWnt8–mFzd8_CRD_–hLRP6_E1E2._ (*B*) 3D reconstruction of haXWnt8–mFzd8_CRD_–hLRP6_E1E2_ colored in purple (haXWnt8), cyan (mFzd8_CRD_), and green (hLRP6_E1E2_). (*C*) Close-up of the Site A and Site B interfaces viewed from the red and blue arrows indicated in the panel *C* *Left*. The cryo-EM map (transparent gray) is superimposed on the ribbon model of the complex (LRP6, green; haXWnt8, purple). The NC-linker shows continuous cryo-EM density, while N-terminal region does not. Map contour levels are set to 0.32 on ChimeraX. The sequence of the engineered haXWnt8 NC-linker is displayed at the *Bottom* of the *Right* panel and on the structure, with the wild-type sequence shown at the *Bottom*. (*D*) LRP6_E1E2_ tetramer binding to hFzd5_CRD_-1TM cells with or without expression of wild-type XWnt8, XWnt8_ΔN_, or XWnt8_ΔNC_, showing that both the N-terminal loop and NC-linker contribute to the LRP6 binding. The tetramer concentration was 50 nM and fourfold lower than the experiment shown in Fig. 1*B*.

To prepare the soluble ternary complex, we cocultured S2 cells expressing haWnt8–mFzd8_CRD_ and hLRP6_E1E2,_ and the complex was purified by a series of affinity and size-exclusion chromatography (SEC) steps. The peak fractions corresponding to the 1:1:1 haXWnt8–mFzd8_CRD_–hLRP6_E1E2_ complex were subjected to cryo-EM analysis (Fig. 2 *A*–*C* and *SI Appendix*, Table S1 and Figs. S3 and S4). Two-dimensional (2D) class averages of the complex revealed the central Wnt bridging the small globular mFzd8_CRD_ and the tandem β-propellers of hLRP6_E1E2_ (Fig. 2*A*). Three-dimensional (3D) reconstruction yielded the 3.8 Å nominal resolution map, revealing the stereotypical Wnt–Fzd_CRD_ binding mode as previously reported (14, 15) (Fig. 2*B*). hLRP6_E1E2_ appears to “cap” XWnt8 at the top of the structure by engaging two sites on XWnt8 distal from the Fzd binding site. Site A is formed by the XWnt8 N-terminal loop, and Site B is formed by the linker between N- and C-terminal domains (NC-linker), which insert into the E1 and E2 funnels in the center of the hLRP6_E1E2_ β-propellers, respectively (Fig. 2*C*). Both binding motifs in XWnt8 were not resolved in the previously reported XWnt8–mFzd8_CRD_ crystal structure (14), suggesting they are disordered in the absence of LRP6.

The flexibility of the unusual interaction mode seen has presumably limited the quality of our cryo-EM map, precluding the placement of amino acid side chains for the XWnt8 N terminus and NC-linker. While the N-terminal interaction is particularly tenuous, the NC-linker mainchain clearly shows that the central region of the loop inserts into the funnel.

The observed Site A interaction mediated by the XWnt8 N terminus was surprising, so we asked whether it was merely an adventitious interaction by virtue of its proximity to the NC-linker interaction with E2. There is precedent for Wnt N termini being important for function in that Tiki family metalloproteases are Wnt inactivating enzymes that cleave N-terminal loops of Wnts (22, 23). We find that deleting the hydrophobic N-terminal loop (XWnt8_ΔN_) reduced LRP6 binding similar to XWnt8_ΔNC_ (Fig. 2*D*). Since the effect was less pronounced than deleting the NC-linker, the NC-linker clearly plays the major role in driving the Wnt–LRP6 association.

### Crystal Structure of the haXWnt8 NC-Linker Peptide Bound to hLRP6_E1E2_.

**Fig. 3.**
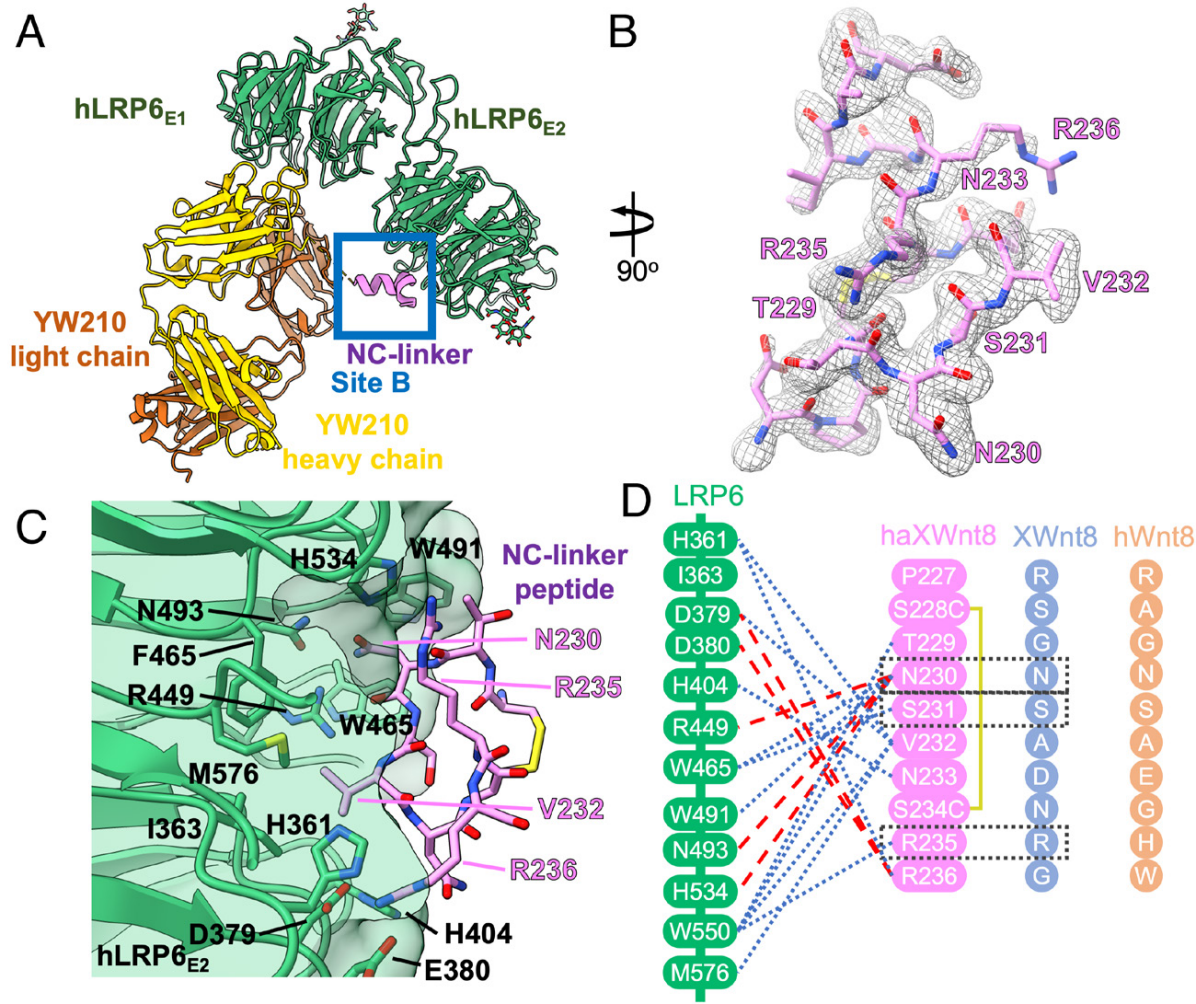
Crystal structure of the YW210-fused haXWnt8 NC-linker in complex with hLRP6_E1E2_. (*A*) Overall view of the crystal structure of hLRP6_E1E2_ (green) bound to YW210 (yellow and wheat) and the haXWnt8 NC-linker (pink). A blue box indicates site B. (*B*) mFo-DFc electron density (gray) from a simulated annealing omit map contoured at 3σ around the NC-linker peptide. (*C*) Close-up view of the boxed region in panel *A*, with the solvent-accessible surface of LRP6 rendered transparently and key interacting side chains labeled; W550 omitted for clarity. (*D*) Alignment of NC-linkers from hWnt8 (orange), XWnt8 (blue), and haXWnt8 (pink), with interactions to LRP6 residues (green) indicated for hydrogen bonds (red dashed line) and van der Waals interactions (blue dashed lines). The engineered disulfide bond in the crystallized NC-linker is indicated by a yellow line. Residues unchanged between XWnt8 and haXWnt8 are boxed.

In order to obtain higher resolution structural data on the NC-linker interaction with the E2 funnel that would enable placement of amino acid sidechains, we took a crystallization-chaperone based approach. We prepared the YW210.09 Fab (named YW210 hereafter), which specifically binds to LRP6_E1_ (17), with the circular NC-linker peptide fused to the N terminus of the heavy chain by a short peptide linker, reasoning that high affinity binding of the Fab to hLRP6_E1_ would place the fused peptide within close proximity to the E2 funnel. The strategy was successful, and we solved this structure to 1.7 Å resolution (Fig. 3*A* and *SI Appendix*, Table S2), revealing clear electron density for the haXWnt8 NC-linker peptide (Fig. 3*B*). The NC-linker binds at the center of the E2 funnel with the buried solvent-accessible surface area of 469 Å^2^ accompanied by substantial structural rearrangements of the E2 β-propeller loops (Fig. 3*C* and *SI Appendix*, Fig. S5*A*). The primary point of contact is N230 of haXWnt8, which binds in a pocket formed by W550^LRP6^, H534^LRP6^, W491^LRP6^, W465^LRP6^, and R449^LRP6^, accounting for 30% of the buried surface area, and forms hydrogen bonds with N493^LRP6^ and H534^LRP6^ (Fig. 3 *C* and *D* and *SI Appendix*, Fig. S5*B*). This residue is conserved in both wild-type XWnt8 and human Wnt8 (Fig. 3*D*). Additional substantial contacts are formed by V232 (alanine in wild-type XWnt8), which binds in a pocket formed by M576^LRP6^, F465^LRP6^, W550^LRP6^, H361^LRP6^, I363^LRP6^, D379^LRP6^, and D406^LRP6^, accounting for an additional 25% of the buried surface area. The G236R mutation of the NC-linker provides additional stabilizing contacts, forming salt bridges with D379^LRP6^ and E380^LRP6^. In short, the crystal structure validates the binding mode of the XWnt8 NC-linker seen by cryo-EM and delineates wild-type versus modified affinity-matured contacts (Fig. 3*D*).

### Generality of NC-Linker to Mammalian Wnts.

**Fig. 4.**
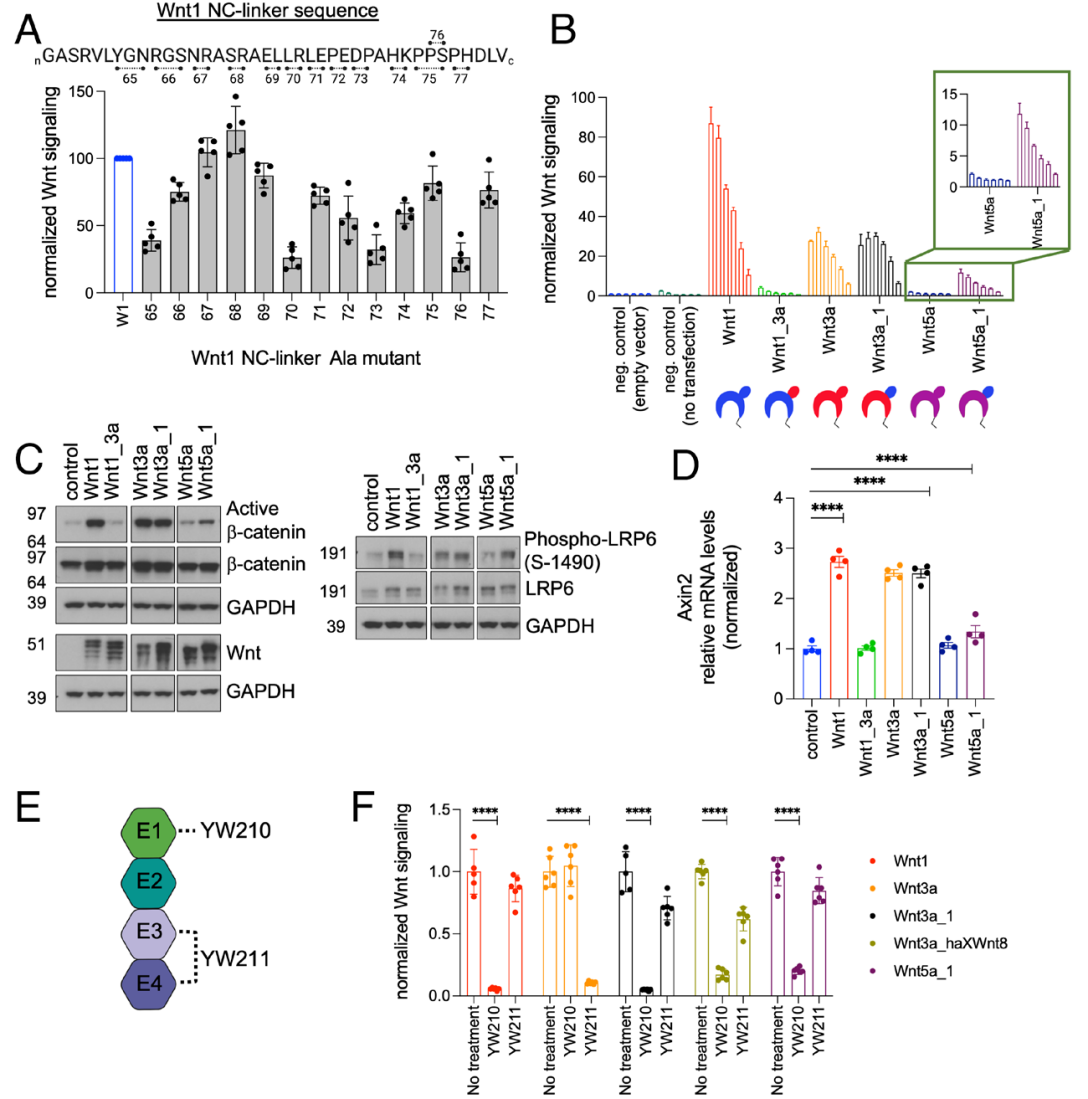
Exchange of the NC-linker between Wnt proteins modifies the signaling characteristics of their parental Wnt. (*A*) Representative TOPbrite dual-luciferase reporter assay showing normalized Wnt signaling induced by various Wnt1 NC-linker alanine mutants from five biological replicates. Sequences are provided in *SI Appendix*, Fig. S5. Bar and error bar represent the mean and SD of five technical replicates. (*B*) Representative TOPbrite dual-luciferase reporter assay measuring Wnt signaling with overexpression of Wnts and Wnt chimeras. Signaling was normalized to the negative control (empty vector). Bar and error bar represent the mean and SD of three technical replicates from one of the three biological replicates. (*C*) Representative Western blot showing β-catenin and LRP6 levels with overexpression of Wnts and Wnt chimeras. (*D*) Quantitative PCR analysis of Wnt target gene *Axin2* with overexpression of Wnts and Wnt chimeras. The expression was normalized to the negative control in each group. Bars represent the means and SEM of four biological replicates. (*E*) Schematic representation of LRP6 domains and domain-specific bound antibodies, YW210 and YW211. (*F*) Representative TOPbrite dual-luciferase reporter assay showing the signaling characteristics of Wnt1, Wnt3a, the Wnt1-mimicking chimeras, Wnt3a_1 and Wnt5a_1, and the haXWnt8-mimicking chimera, Wnt3a_haXWnt8. The signaling was normalized to the negative control (without any treatment) in each group. Bar and error bar represent the means and SD of six technical replicates. ****P < 0.0001, one-way ANOVA with Tukey’s multiple comparison test from one of the three biological replicates. Data were analyzed with Prism 9.

The NC-linker is one of the least conserved amino acid stretches across different Wnt proteins, but it is highly conserved across species for a particular Wnt (*SI Appendix*, Fig. S6). Using human Wnt1 as a model system, we performed alanine scanning mutagenesis on the NC-linker analogous to that of XWnt8 to assess its functional role in mediating Wnt/β-catenin signaling. Despite comparable expression, most Wnt1 variants were less active in inducing β-catenin signaling compared to wild-type Wnt1, with mutants 65, 70, 73, and 76 having the most impaired signaling activities (Fig. 4*A* and *SI Appendix*, Fig. S7). Thus, the NC-linker of human Wnt1 also appears to be a critical mediator of Wnt signaling activity.

### The NC-Linker Controls Wnt–LRP6 Domain Specificity.

To investigate the modularity of the NC-linker in mediating LRP6 interactions by different Wnts, we designed three chimeric Wnt constructs in which the NC-linker was swapped between different human Wnt proteins (Fig. 4*B*). We chose Wnt1, Wnt3a, and Wnt5a as templates for NC-linker swapping because they represent canonical (Wnt1 and Wnt3a) and non-canonical (Wnt5a) Wnts and interact with LRP6_E1E2_ (Wnt1) and LRP6_E3E4_ (Wnt3a) (16, 17). Since no structures of Wnt1 and Wnt5a were available, we expected that the structural imprecisions of the NC-linker grafts in the chimeric molecules will quantitatively influence their activities. With these caveats in mind, we undertook a range of grafts and assessed their activities. As expected, the canonical Wnt proteins Wnt1 and Wnt3a induced β-catenin signaling in TOPbrite assays, whereas the non-canonical Wnt5a did not induce reporter expression above background (24) (Fig. 4*B*). Grafting the NC-linkers from the E1E2-binding Wnt1 and haXWnt8 into the E3E4-binding Wnt3a (termed Wnt3a_1 and Wnt3a_haXWnt8) did not markedly alter the activity of wild-type Wnt3a, suggesting that we successfully transferred LRP6_E1E2_ specificity to Wnt3a from Wnt1 and haXWnt8, respectively (Fig. 4*B* and *SI Appendix*, Fig. S8*A*). However, the reverse engineered Wnt1_3a chimera was inactive. Grafting the Wnt3a NC-linker may have less tolerance for different structural contexts. Consistent with this, grafting the Wnt1 NC-linker into Wnt5a rendered the Wnt5a_1 chimera able to modestly induce β-catenin signaling (Fig. 4*B*), functionally turning a non-canonical Wnt into a canonical Wnt. Consistently, Wnt1, Wnt3a, Wnt3a_1, and Wnt5a_1 but not Wnt5a and Wnt1_3a induced LRP6 phosphorylation, the accumulation of active (nonphosphorylated) β-catenin (Fig. 4*C* and *SI Appendix*, Fig. S8*B*), and Axin2 transcription (Fig. 4*D*), all characteristic hallmarks of canonical Wnt signaling. Grafting the Wnt3a NC-linker into Wnt5a (Wnt5a_3a) did not result in activity.

To further interrogate the LRP6_E1E2_ versus LRP6_E3E4_ binding specificity of the Wnt chimeras, and the role of the NC-linker in determining binding preferences, we leveraged two LRP6 domain specific antibody fragments (Fab): Fab YW210 binds predominantly to LRP6 E1 domain, and Fab YW211.31.62 (named YW211 hereafter) binds to LRP6 E3E4 domain (17, 25) (Fig. 4*E* and *SI Appendix*, Fig. S8*C*). As expected, YW210 inhibited Wnt1-induced signaling and YW211 inhibited Wnt3a-induced signaling in luciferase assays, consistent with the reported LRP6 domain specificity (Fig. 4*F* and *SI Appendix*, Fig. S8*D*). However, the activities of the Wnt3a_1, Wnt3a_haXWnt8, and Wnt5a_1 chimeras were inhibited by YW210, and not by YW211, demonstrating that the Wnt1 and haXWnt8 NC-linkers direct the binding of these chimeras to LRP6_E1E2,_ mimicking Wnt1 and XWnt8 signaling characteristics, respectively. In summary, these results suggest a critical role of the Wnt NC-linkers in determining canonical and non-canonical Wnt signaling activity and LRP6 domain specificity.

### Synthetic Peptides Mimicking NC-Linker Inhibit Wnt Activity.

**Fig. 5.**
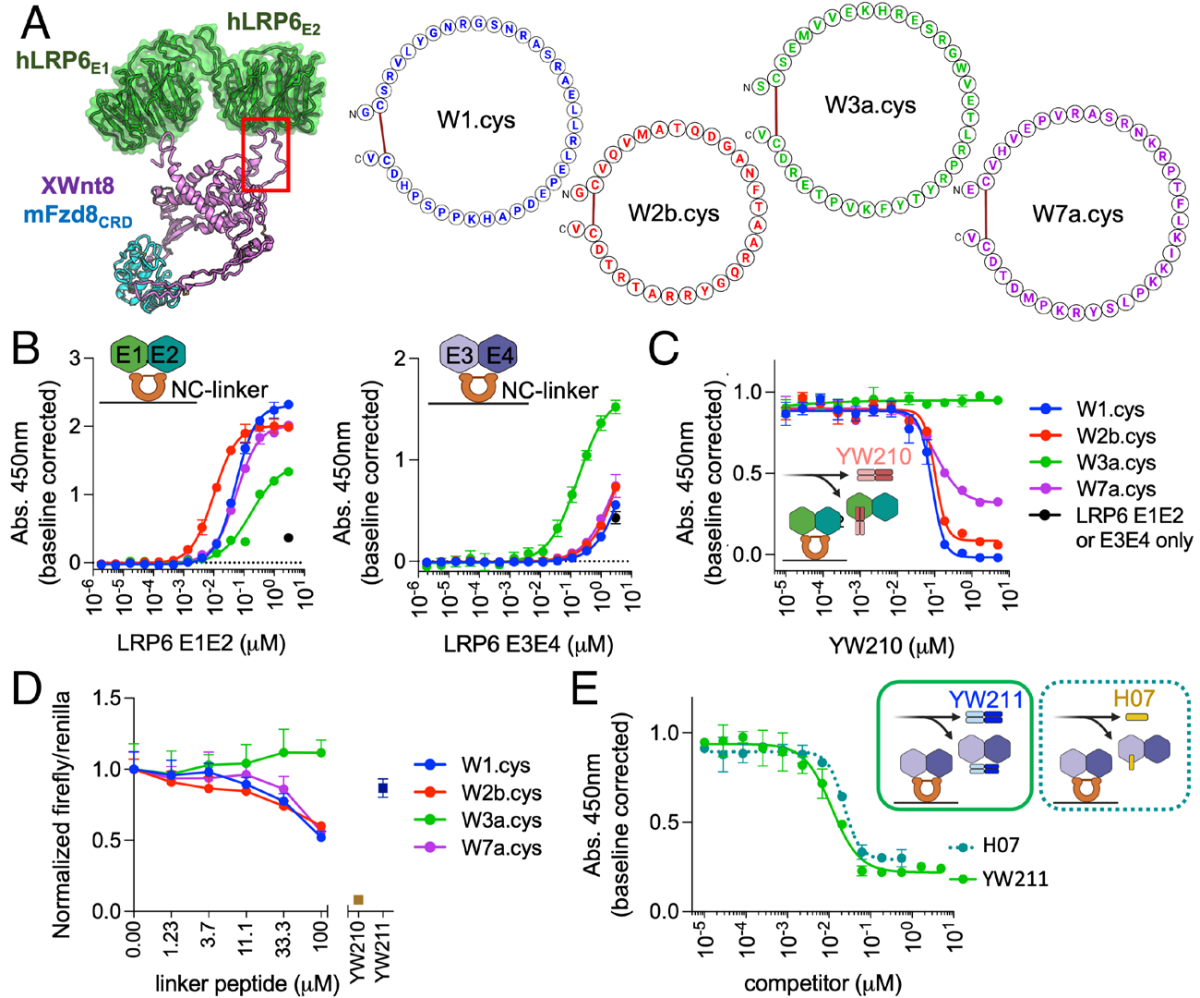
Synthetic Wnt linker peptides show LRP6 domain specific binding. (*A*) A ribbon-and-surface model of haXWnt8–mFzd8_CRD_–hLRP6_E1E2_ colored in purple (haXWnt8), cyan (mFzd8_CRD_), and green (hLRP6_E1E2_) (*Left*). The sequences of W1.cys, W2b.cys, W3a.cys, and W7a.cys are portrayed in the schematic form showing the constrained peptides by a disulfide bond (*Right*). (*B*) Representative ELISA binding assay of Wnt.cys peptides to LRP6_E1E2_ (*Left*) and LRP6_E3E4_ (*Right*). The signals from the binding of various Wnt.cys peptides to the assay plate were subtracted from their binding signals to LRP6. Data are shown as the means with SD of three technical replicates from one of the three biological replicates. (*C*) Representative ELISA competition assay of LRP6 E1E2-binding Wnt.cys peptides with Fab YW210. (*D*) Dose-dependent inhibition of Wnt1-mediated signaling by W1.cys, W2b.cys, and W7a.cys. Data are shown as the means with SD of three technical replicates from one of the three biological replicates. (*E*) Representative ELISA competition assay of LRP6 E3E4-binding Wnt3a.cys with Fab YW211 and H07 VHH. Only the peptides showing binding to each functional domain of LRP6 were tested in the competition ELISA. Data are shown as the means with SD of three technical replicates from one of the three biological replicates. Data were analyzed with Prism 9.

We investigated the binding specificities of isolated NC-linker peptides and their ability to interfere with Wnt activity. Superposition of the three available wild-type Wnt structures (14, 15, 21) revealed two loosely structurally conserved “interface motifs (15, 26)” that flank the variable NC-linkers (*SI Appendix*, Fig. S9 *A* and *B*). We designed cyclic peptides mimicking the NC-linkers (termed W.cys) of four different Wnts and engineered cysteine residues at two conserved positions within the interface motifs that are within optimal distance (Cβ–Cβ distance of ~4.0 Å) for disulfide bond formation (Fig. 5*A* and *SI Appendix*, Fig. S9*B*). In an enzyme-linked immunosorbent assay (ELISA) experiment, we observed binding of W1.cys, W2b.cys, and W7a.cys to LRP6_E1E2_ (Fig. 5 *B*, *Left*), and of W3a.cys to LRP6_E3E4_ (Fig. 5 *B*, *Right* and *SI Appendix*, Table S3). This is consistent with previously reported Wnt–LRP6 domain specificity (17). We further demonstrated that W7a.cys, but none of the other NC-linker peptides, bound Reck (*SI Appendix*, Fig. S10*A*), consistent with an earlier report (27). The weaker interaction between W3a.cys and LRP6_E1E2_ can be attributed to “sticky” nonspecific binding as it could not be outcompeted by anti-LRP6_E1_ YW210 (Fig. 5*C*). Despite the inherent flexibility of isolated peptides, W1.cys, W2b.cys, and W7a.cys, but not W3a.cys, inhibited Wnt1-mediated β-catenin signaling in luciferase assays without any observed toxicity on cells (Fig. 5*D* and *SI Appendix*, Fig. S10*B*), presumably by competing with Wnt1 for LRP6_E1E2_ binding. The lack of activity of the Wnt3a peptide is consistent with its binding ability to LRP6_E3E4_. Taken together, the overall consistency in LRP6 domain specificity of isolated NC-linker peptides and full-length Wnt proteins, as well as the ability of the peptides to interfere with Wnt activity, further supports the critical role of the NC-linker in determining LRP6 domain specificity.

To further map the binding region of the NC-linker peptides on LRP6, we performed a competition ELISA with YW210, YW211, and H07, a variable domain of heavy chain of heavy-chain antibody (VHH) that binds to the LRP6 E3 domain (28). YW210 competed strongly with W1.cys and W2b.cys, and partially with W7a.cys for LRP6_E1E2_ binding (Fig. 5*C*). Given the relatively large size of Fabs, it likely sterically masked neighboring binding epitopes on LRP6_E2_. Furthermore, YW211 and H07 VHH both competed with W3a.cys for binding to LRP6_E3_ (Fig. 5*E*), suggesting that the Wnt3a NC-linker binds to the E3 domain of LRP6.

**Fig. 6.**
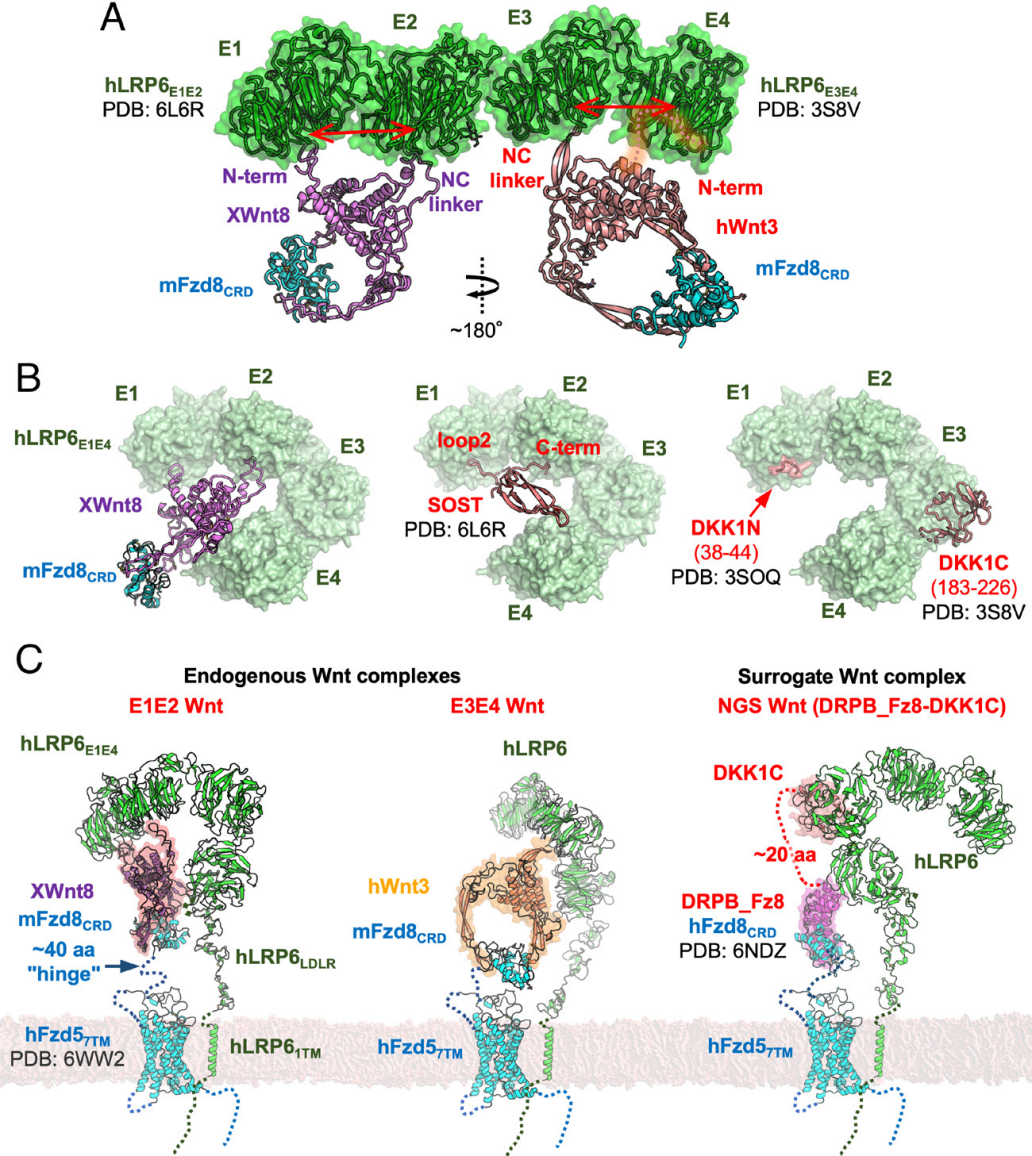
Modeling initiation and inhibition of Wnt signaling. (*A*) Binding modes of E1E2-binding Wnt8 and E3E4-binding Wnt3 to LRP6. Crystal structures of LRP6_E1E2_ and LRP6_E3E4_ are placed linearly, and mFzd8_CRD_–XWnt8 and mFzd8_CRD_–hWnt3 are anchored to the propeller funnels in the orientation suggested by the competition assays. LRP6, mFzd8, XWnt8, and hWnt3 are colored in green, cyan, purple, and pink, respectively. (*B*) The structural models of hLRP6_E1E4_ binding to haXWnt8–mFzd8_CRD_ (*Left*), sclerostin (*Center*), or DKK1 (*Right*). (*C*) Schematic for the transmembrane LRP6–Fzd5 signaling complex with endogenous Wnts or Surrogate Wnt (10).

hLRP6_E1E2_ and hLRP6_E3E4_ are relatively conserved regarding their funnel-like cavity at the center of the β-propellers, hydrophobicity and electrostatic property of β-propellers, and the ~40 Å distance between the proximal funnels (*SI Appendix*, Fig. S11), consistent with a shared two-site Wnt binding mechanism by both LRP6_E1E2_ and LRP6_E3E4_. Our H07 VHH blocking data show that E3 domain blockade prevents Wnt3a binding, localizing the NC-linker to the LRP6 E3 domain, while the NC-linker of XWnt8 and Wnt1 binds to the LRP6 E2 domain. This structure–function data now allow us to orient how E1E2-binding versus E3E4-binding Wnts engage LRP6 through their NC-linkers and N termini. We propose that, like XWnt8, E1E2-binding Wnts use their N termini to bind E1 and NC-linker to bind E2, whereas E3E4-binding Wnts, represented by Wnt3, use their N termini to bind E4 and their NC-linker to bind to E3. Thus, E1E2-binding versus E3E4-binding Wnts appear to bind to LRP6 domains in “reverse” orientations to accommodate the NC-linker specificity (Fig. 6*A*).

## Discussion

The structure of the ternary haXWnt8–mFzd8_CRD_–hLRP6_E1E2_ complex resolves the basis for coreceptor binding by Wnts, revealing a modular determinant that not only controls LRP5/6 domain specificity, but also appears to differentiate the non-canonical Wnt5a and explain its coreceptor independence. The signaling complex structure further elucidates the mechanism of LRP5 gain-of-functions mutations (including D111Y, R154M, G171V/R, N198S, A214V/T, and T253I which correspond to D98, R141, G158, N185, A201, and T240 in LRP6) that are associated with high bone mass (HBM) disease and the significant increase in bone strength and thickness in affected patients (29, 30). These HBM mutations are located on the top surface of the E1 β-propeller of LRP5, and overlap with the binding epitopes of Wnt antagonists sclerostin (SOST) (31) or Dickkopf (DKK) (32, 33) (Fig. 6*B*). Consistent with the notion that the Wnt NC-linker–LRP6_E2_ interaction is the main driver for binding, HBM mutations impaired the binding of SOST and DKK1, but with a minimal effect on Wnt9b in functional assays, resulting in the selective loss in affinity for Wnt signaling antagonists (25, 34, 35).

The ECD of LRP5/6 consists of four YWTD β-propellers flanked by EGF-like domains, E1 to E4 (Fig. 6 *B*, *Left*), followed by three low-density lipoprotein receptor type A domains, and exists in a range of bend conformations (36). The ECD interacts with Wnt proteins and antagonists, including SOST and DKK, to regulate signaling. While SOST antagonizes LRP6_E1E2_-binding Wnts including Wnt1, Wnt2, and Wnt9b, but not LRP6_E3E4_-binding Wnts including Wnt3a (37), DKK1 inhibits both the Wnt groups (16, 38). SOST engages the top surfaces of the E1 and E2 β-propellers with its loop 2 and the C-terminal tail (25, 37, 39), respectively, reminiscent of the tandem binding mode of haXWnt8. SOST occludes both Sites A and B of the haXWnt8–LRP6 interfaces (Fig. 6 *B*, *Middle*), demonstrating that SOST antagonizes Wnt signaling predominantly through the direct competition with Wnts for LRP6 binding. This competitive tandem binding mode of SOST and Wnt is further supported by functional studies showing that both loop 2 and the C-terminal tail of SOST are critical for inhibiting β-catenin signaling mediated by Wnt1, Wnt2, and Wnt9b (37).

DKK comprises another class of four broadly expressed Wnt antagonists (32, 33). The N- and C- terminal fragments of DKK1, DKK1N (25), and DKK1C (40) bind to the funnel surface of the LRP6 E1 and E3 β-propellers, respectively (Fig. 6 *B*, *Right*). The binding site of DKK1N overlaps with that of the Wnt N-terminal loop on E1, but not with the binding site of the NC-linker on E2, which is the major driver of the Wnt–LRP6 interaction. Upon binding of DKK1 to LRP6, the LRP6 ECD adopts a compact conformation, bringing the E1E2 and E3E4 domains into close proximity in a face-to-face orientation (36). Together with our structure, this demonstrates that DKK1 inhibits Wnt binding to LRP6_E1E2_ and LRP6_E3E4_ by directly competing for LRP6 binding as well as through steric occlusion of Wnts from the compact ECD fold.

The ternary XWnt8–mFzd8_CRD_–hLRP6_E1E2_ complex structure, together with the structure of the Fzd transmembrane regions (41, 42), and previous mechanistic insights into heterodimeric Wnt activation (10, 42) allow us to propose a structural model of the transmembrane Wnt signaling complex for both LRP6 E1E2 and E3E4 binding Wnts (Fig. 6*C*). Given the different topologies of these signaling complexes, it is tempting to speculate that this could affect signaling strength. We have recently shown that bispecific Wnt “surrogates” composed of an Fzd_CRD_ binding domain fused to DKK1C can activate β-catenin signaling by heterodimerizing Fzd and LRP6. However, the surrogates with different binding domains and linkers show great variations in signaling strength, suggesting that the geometry (i.e., orientation and proximity) of the two receptor chains within the signaling complex is a critical determinant of signaling characteristics. Furthermore, the geometry of the signaling complex induced by the Wnt surrogates and natural Wnt ligands differs greatly, possibly yielding the differential E_max_. Thus, the haWnt8–mFzd8_CRD_–hLRP6_E1E2_ structure provides a blueprint to rationally improve the Wnt surrogates by modulating receptor complex geometry as has been done in homo- and heterodimeric cytokine receptors (43, 44), facilitating more effective Wnt surrogates for regenerative medicine.

## Materials and Methods

### Mammalian Cell Line for Wnt Display.

To display functional Wnts on cells, we engineered HEK293F cells (Thermo Fisher) to saturate the cell surface with hFzd5_CRD_ (residues 27 to 155) in a manner analogous to scFv displayed on mammalian cells (19). The N-terminal HA-tagged hFzd5_CRD_ was fused to the membrane-proximal region and transmembrane domain of gorilla platelet-derived growth factor receptor-β (PDGFR-β, residues 513 to 561) with a linker containing Myc tag (PG**SGEQKLISEEDL**GNGNGNGNGNGNGNGNGNGNGNG), and cloned into the lentiviral gene ontology vector which has EF-1α promoter and Igκ signal peptide (45), termed pTether hFzd5_CRD_-1TM. For lentivirus packaging, Lenti-X 293T cells (Takara Bio) were transfected with pTether hFzd5_CRD_-1TM, pMD2.G (Addgene 12259), and psPAX2 (Addgene 12260) vectors using FuGENE HD (Promega), and the cells were cultured at 37 °C for 3 d. Lenti-X 293T cells were maintained in Dulbecco’s Modified Eagle Medium (DMEM, Gibco) supplemented with 10% (v/v) fetal bovine serum (FBS, Sigma) and GlutaMAX (Gibco), or “complete DMEM,” at 37 °C and 5% CO_2_. The media was exchanged with FreeStyle 293 media (Thermo Fisher) prior to the transfection, and the supernatant containing the lentivirus was harvested and used immediately to infect HEK293F cells maintained in FreeStyle 293 media at 37 °C and 5% CO_2_ with gentle agitation. After 3 d, cells were stained using Alexa Fluor 647 Conjugated HA-Tag (6E2) Mouse mAb (Cell Signaling 3444) and analyzed by an Accuri C6 Plus flow cytometer (BD Bioscience) to check the surface hFzd5_CRD_ expression, and high-expression clones were bulk sorted by FACS using SH800S Cell Sorter (Sony Biotechnology).

### Wnt Display.

To test Wnt display on the cell surface, *Xenopus laevis* Wnt8 (XWnt8) was expressed in the parental HEK293F cells and the hFzd5_CRD_-1TM cells before FACS sorting. Full-length XWnt8 was cloned into pcDNA3.1+ (Thermo Fisher) vector with endogenous signal peptide and C-terminal Protein C epitope. The cells were transiently transfected with the pcDNA3.1+ XWnt8 vector and stained by Alexa Fluor 647 conjugated Protein C antibody (PrC647; the antibody produced in-house from HPC-4 hybridoma, ATCC HB-9892, and labeled with Alexa Fluor 647 NHS Ester, Thermo Fisher), showing superior Wnt display on the hFzd5_CRD_-1TM cells. The flow cytometry data were analyzed using FCS Express 7 (De Novo Software).

To confirm that the lipidated Wnt stays on the cell which produced the molecule, the sorted hFzd5_CRD_-1TM cell line was transfected either with the XWnt8 or green fluorescent protein (GFP)-coding mammalian expression vector, and 24 h after transfection, the Wnt- and GFP-induced cells were mixed at 1:1 ratio and further shaken at 37 °C for 24 h. Cell-surface Wnt was labeled with PrC647, and Wnt/GFP expression was checked by the flow cytometry. Wnt transfer to the GFP positive cells was estimated to be ~5%.

To prepare stable cell lines expressing XWnt8 or hWnt8 with or without the deletion of the NC-linkers (residues 222 to 234 for XWnt8, and 221 to 233 for hWnt8, that were replaced by a Gly-Ser-Gly-Ser linker), and N-terminal loops (residues 23 to 32 for XWnt8), the constructs were cloned into the pLV-EF1a-IRES-Puro vector (Addgene 85132) with N-terminal HA signal peptide and C-terminal FLAG tag. The lentiviruses were prepared and added to the hFzd5_CRD_-1TM cell line, and 2 d after infection, the transduced cells were selected by adding 1 μg/mL puromycin (Gibco) and incubated for 2 more days. Wnt display was confirmed by mouse monoclonal ANTI-FLAG M2-FITC antibody (FLAG-FITC, Sigma F4049), showing comparable display between the full-length and NC-linker truncated versions.

### Preparation of Recombinant hLRP6_E1E2_ for Cell Staining.

Like the majority of Wnts, XWnt8 and hWnt8 bind to the E1E2 module of human LRP6 (hLRP6_E1E2_). To recombinantly express hLRP6_E1E2_ and check Wnt8-hLRP6_E1E2_ interaction, hLRP6_E1E2_ (residue 20 to 629) was cloned into pAcGP67A (BD Biosciences) vector with C-terminal Biotin Accepter Peptide (BAP) and hexahistidine tags. *Spodoptera frugiperda* (Sf9) ovarian cells (ATCC CTL-1711) were maintained in Sf-900 III medium (Gibco) with 10% (v/v) FBS and GlutaMAX at 27 °C with ambient CO_2_ and gentle agitation. P0 virus was produced by transfection with FuGENE HD and BestBac 1.0 Linearized Baculovirus DNA (Expression Systems) followed by a viral amplification to P1. The protein was expressed in *Trichoplusia ni* ovarian cells (Expression Systems) for 60 to 72 h maintained in ESF 921 Insect Cell Culture Medium (Expression Systems) at 27 °C with ambient CO_2_ and gentle agitation. The protein was purified from culture supernatant with Ni-NTA (Qiagen), biotinylated with in-house purified BirA enzyme, purified over a Superdex 200 10/300 GL size-exclusion chromatography (SEC) column (Cytiva), and equilibrated with HBS (10 mM HEPES-Na and 150 mM NaCl) and 2 mM CaCl_2_. The peak fraction was concentrated to ∼30 μM, aliquoted, and flash frozen. A frozen aliquot was thawed and mixed with Streptavidin (Thermo Fisher) for sodium dodecyl sulfate polyacrylamide gel electrophoresis (SDS-PAGE) bandshift assay to confirm ~100% biotinylation.

### hLRP6_E1E2_ Tetramer Staining of the Wnt-Expressing Cells.

Biotinylated hLRP6_E1E2_ was tetramerized with Alexa 647 labeled streptavidin (SA647, streptavidin with a cysteine residue produced in house, and labeled with Alexa Fluor 647 C2 Maleimide, Thermo Fisher) and cells were stained with 30 to 200 nM hLRP6_E1E2_-SA647 tetramer (120 to 800 nM hLRP6_E1E2_ as monomer). The stained cells were washed with PFE (PBS, 2% (v/v) FBS, 2 mM EDTA) twice, and analyzed with the flow cytometer. The initial tests were performed with transient Wnt expression, and the tetramer staining was further confirmed for stable XWnt8 and hWnt8 cell lines described above. Clear tetramer staining was observed for the wild-type Wnt8s, but as suggested for hWnt3a (15, 21), the NC-linker deletion decreased hLRP6_E1E2_ binding to Wnt8s (Fig. 1*B* and *SI Appendix*, Fig. S2), underscoring the premise for library creation on this linker. The flow cytometry data were analyzed using FCS Express 7.

### XWnt8 Library Construction.

After confirming that the Wnt8s' NC-linker mediates interaction with hLRP6_E1E2_, two XWnt8 libraries were constructed on the linker targeting 1) family conserved amino acid residues and 2) other amino acid residues (*SI Appendix*, Fig. S1*A*). In the parental pLV-EF1a-IRES-Puro XWnt8 plasmid, the XWnt8 gene was codon optimized, and restriction enzyme sites were introduced at around E201 (EcoRI) and G247 (BamHI) with silent mutations (*SI Appendix*, Fig. S1*A*). The library genes were ordered from Eurofins Scientific as EXTREmers Long DNA and amplified by PCR for joining with the XWnt8 vector cut by the EcoRI and BamHI enzymes (New England Biolabs, NEB). Each library insert and the cut XWnt8 vector were mixed at 1:2 molar ratio, and the DNAs were assembled by NEBuilder HiFi DNA Assembly Master Mix (NEB) and purified with Zymo-Spin I Columns (Zymo Research) for electroporation to *Escherichia coli*. The purified DNA was added to MegaX DH10B T1R Electrocomp Cells (Thermo) on ice, and the cells were placed in the ice-cold electroporation cuvettes (2 mm gap, Fisher Scientific) for transformation with MicroPulser Electroporator (BioRad). The cells were incubated with the provided recovery medium before plating. The efficiency was checked with serial dilution on Lysogeny broth (LB) plates supplemented with 100 μg/mL carbenicillin, and according to the titration result, the freshly electroporated and recovered *E. coli* libraries were plated on eight each of LB square bioassay dishes (Corning) supplemented with 100 μg/mL carbenicillin and incubated at 37 °C overnight. Approximately 0.5 million transformants were obtained for each library. The *E. coli* colonies were collected with scrapers and the library plasmids were purified using Plasmid Maxi Kit (Qiagen). Lentiviral libraries were prepared as described above in complete DMEM and harvested using PEG-it Virus Precipitation Solution (System Biosciences). Lentiviral titers were checked using the hFzd5_CRD_-1TM cells at 2 mL scale based on Wnt expression, and 120 mL of 1 × 10^6^ cells/ml was used to have ~1% cells infected. After 3 d of infection, the cells were stained by FLAG-FITC and the transduced cells were isolated by magnetic-activated cell sorting using anti-FITC MicroBeads (Miltenyi) and LS columns (Miltenyi). The library cells were grown in FreeStyle 293 media, and once the total cell number reached >10 million for both library, 5 million cells each of FLAG-FITC+ library 1 and 2 were mixed to make the naïve XWnt8 library.

### XWnt8 Library Selection and the NC-Linker Chimera of hWnt8a.

The naïve XWnt8 library was stained with 100 nM hLRP6_E1E2_-SA647 tetramer and 1:50 FLAG-FITC at 4 °C for 1 h, washed twice with PFE, passed through 70 μm Nylon Sterile Cell Strainers (Corning), and sorted using SH800S Cell Sorter. Top 7 to 8% clones based on XWnt8 expression (FLAG-FITC) and hLRP6_E1E2_ binding (SA647) were selected, then washed with and re-grown in FreeStyle 293 media. The following rounds of selections were performed in the same manner as the first round with increased stringency: The second selection was performed with 50 nM hLRP6_E1E2_ tetramer and the selection of top 7 to 8% clones, and the final, third round was done with 30 nM hLRP6_E1E2_ tetramer and ~5% gating (*SI Appendix*, Fig. S1*B*). hLRP6_E1E2_ tetramer staining showed increased binding during the selection (Fig. 1*C*). The genomes were then extracted from the post-round three selection library using QIAmp DNA Mini Kit (Qiagen), and the library regions on the Wnt gene were amplified by PCR and recloned into the pLV-EF1a-IRES-Puro vector for sequencing and binding experiments. The single-clone Wnt variants were introduced to the hFzd5CRD-1TM cells and hLRP6_E1E2_ binding was confirmed on the Wnt-expressing cells. All the five single clones tested (named haXWnt8, haXWnt8a, haXWnt8b, haXWnt8c, haXWnt8d) showed improved binding to hLRP6_E1E2_ compared to the wild-type XWnt8 (Fig. 1*D* and *SI Appendix*, Fig. S2).

The engineered NC-linkers from the XWnt8 library were grafted to hWnt8 to test whether they improve the hLRP6_E1E2_ binding. The wild-type pLV-EF1a-IRES-Puro hWnt8 plasmid was modified to have the engineered NC-linkers from haXWnt8, haXWnt8c, and haXWnt8d synthesized by Integrated DNA Technologies, Inc., making hahWnt8, hahWnt8c, and hahWnt8d, respectively. The hahWnt8s-expressing cell lines were prepared and hLRP6_E1E2_ tetramer staining was confirmed as described above in comparison with the wild-type hWnt8, showing superior binding (Fig. 1*D* and *SI Appendix*, Fig. S2).

The hLRP6_E1E2_ monomer binding was confirmed by incubating the hFzd5_CRD_-1TM cells that display Wnt8 variants with biotinylated hLRP6_E1E2_ at 30, 100, 300, 1,000, 3,000 nM concentrations, followed by washes with PFE and labeling with 50 nM SA647 (binding) and 1:50 FLAG-FITC (Wnt expression). The cells were again washed with PFE twice and analyzed by the flow cytometer. Two independent experiments were performed in triplicate, and the representative experiment was shown with Prism 9 (GraphPad).

### Preparation of Recombinant XWnt8 and haXWnt8s.

The five haXWnt8 variants (residues 22 to 338) were cloned into the pActinCD8 vector as previously described (14). *Drosophila* S2 cells were maintained in Schneider’s *Drosophila* Medium (Gibco) supplemented with 10% (v/v) FBS and GlutaMAX at 27 °C with ambient CO_2_. The cells were cotransfected with wild-type or variants XWnt8, the pActinCD8 mFzd8_CRD_-Fc vector (14), and pCoBlast using the calcium phosphate precipitation kit (Invitrogen) according to the manufacturer’s protocol. Each cell line was bulk selected for 3 wk with a gradual increase of Blasticidin (Invitrogen) concentrations; 5 μg/mL, 10 μg/mL, 20 μg/mL, for the first, second, and the third week, respectively. Expression levels were checked by Western blotting using Rabbit *X. laevis* Protein Wnt-8 Polyclonal Antibody (MyBioSource, MBS1497175) as a primary antibody and Dako Rabbit Anti-Goat Immunoglobulins/horseradish peroxidase (Agilent, P0449) as a secondary antibody, where haXWnt8 showed the best expression while haXWnt8d had substantial degradation. After the selection, the haXWnt8 cell line was transferred to conical flasks for gentle agitation, and when the culture volumes reached ~50 mL, the cells were gradually expanded in shaker flasks with ESF 921 Insect Cell Culture Medium supplemented with 20 μg/mL Blasticidin to a final volume of 1L and incubated for 5 d.

XWnt8 and haXWnt8 were purified from clarified culture supernatants (8, 42). The XWnt8–mFzd8_CRD_–Fc complex was captured by rProtein A Sepharose Fast Flow (Cytiva). After washing the sepharose resin with 10 column volumes HBS, XWnt8 was eluted with HBS containing 0.1% (v/v) n-dodecyl β-D-maltoside (DDM, Anatrace, Sol-Grade) and 500 mM NaCl, while the mFzd8_CRD_-Fc remained bound to the resin. Wnts were further purified by Con A agarose (42).

### TOPflash Signaling Assay.

To confirm the affinity-enhancing mutations do not interfere with XWnt8 activity, SuperTopFlash HEK293 (293STF) cells were stimulated with a gradient concentration of haXWnt8, wild-type XWnt8, and next-generation surrogate (NGS) Wnt in the presence or absence of R-spondin 2 (8, 10). The agonist concentrations were 0.39 nM to 50 nM for XWnt8s and 0.016 nM to 2 nM for NGS Wnt, respectively, with two-fold serial dilution. 293STF were seeded into 96-well plates and maintained in DMEM media supplemented with 10% (v/v) FBS 1 d before Wnt stimulation. Cells were stimulated on the next day and lysed after 24 h following manufacturer’s protocol in Dual Luciferase Assay kit (Promega). Luminance signal readings were performed using SpectraMax i3x (Molecular Devices). Two independent experiments were performed in triplicate, and the representative experiment was plotted and analyzed by Prism 9.

### Preparation of the Ligand–Receptor Complex for Structural Study.

The S2 cell line expressing hLRP6_E1E2_ was prepared as described above with the pActinCD8 and pCoBlast vectors. The S2 haXWnt8–mFz8_CRD_–Fc cells and hLRP6_E1E2_ cells were mixed at the cell ratio of 1:3, and cocultured for 5 d. The complex was captured by rProtein A Sepharose, and eluted by adding in-house 3C protease, while the cleaved Fc remained bound to the resin and hLRP6_E1E2_ was affinity-purified by haXWnt8 bound to mFzd8_CRD_. The elution was concentrated down to 500 μL and injected to a Superdex 200 10/300 GL column equilibrated with HBS containing 2 mM CaCl_2_. The peak fraction with a retention volume corresponding to the 1:1:1 complex was concentrated to 0.5 mg/mL for cryo-EM analysis.

### Cryo-EM Specimen Preparation and Data Collection.

3 µL sample was applied onto glow-discharged 300 mesh gold grids (UltrAuFoil R1.2/1.3). Excess sample was blotted to a filter paper for 3 s with botting force of 3, before plunge-freezing with a Vitrobot Mark IV (Thermo Fisher Scientific) at 4 °C and 100% humidity. The cryo-EM movies were collected using a Titan Krios (Thermo Fisher Scientific) operated at 300 kV equipped with Gatan K3 camera in counting mode. Nominal magnification was set to 29,000×, corresponding to a pixel size of 0.8521 Å and a calibrated magnification of 58,680×. Movies were recorded using SerialEM (46) for 2.5 s with 0.05 s exposure per frame at an exposure rate of ~16 electrons/pixel/sec at the specimen, and the nominal defocus range between −1.0 and −2.0 μm. A beam-image shift was used with an active calibration to collect nine movies from nine holes per stage shift and autofocus.

### Cryo-EM Data Analysis.

Collected cryo-EM movies were processed and assessed with cryoSPARC Live. Patch motion correction was performed for the movies with a native pixel size of 0.8521 Å, and patch contrast transfer function (CTF) parameters were estimated with a default setting. 10,077 curated movies were used for the downstream processing by cryoSPARC (47) (*SI Appendix*, Fig. S4*B*). Particles were picked with 2D templates from preliminary 2D classification and extracted with a box size of 352 pixels, binned to 132 pixels. Multiple rounds of 2D classifications, multi-class ab intio 3D reconstructions, and nonuniform 3D refinements yielded a preliminary 3D reconstruction with 43,337 particles. Using this particle set, three rounds of topaz (48) picking and extraction were performed with a default setting using 918 micrographs and 50 to 200 expected particles per micrograph for training. This yielded a total of 3,840,364 particles including duplicated particles with the 352/132 binned box. After 2D classifications, multi-class ab initio 3D reconstruction, and removing duplicates with 20 Å minimum separation distance, 276,237 particles were extracted with a box size of 352 pixels without binning. Further 2D classifications and ab initio 3D reconstructions identified 82,235 particles which were reextracted with the 352-pixel box without binning, and beamtilt was fitted by Global CTF Refinement for each image shift group. Local 3D nonuniform refinement was performed using the mask covering the whole complex. The map was sharpened by deepEMhancer (49) for visualization. The reported nominal resolution was 3.8 Å as determined by the 0.143 gold-standard FSC cutoff using 3DFSC (50). As expected from the 2D class averages with preferred particle orientation (Fig. 2*A* and *SI Appendix*, Fig. S4*C*), the map has a large variation in the directional resolutions, as well as highly diverged local resolutions, with the LRP6_E1E2_ density better defined (*SI Appendix*, Fig. S4 *D*–*F*). The 3D map also indicates exposure to the air–water interface around XWnt8’s lipid (*SI Appendix*, Fig. S4*E*).

### Cryo-EM Model Building and Refinement.

The crystal structures of XWnt8-mFzd8_CRD_ (PDB 4F0A) and hLRP6_E1E2_ (PDB 3S94) were manually placed into the cryo-EM map using UCSF ChimeraX (51). To facilitate map interpretation beyond the docking model, the missing N-terminal loop and NC-linker were modeled in the cryo-EM density using Coot (52). Clearly distorted regions from the initial model, represented by the Wnt index finger, were flexibly fitted. We note that, based on the docked initial structures, the regions with weak cryo-EM density such as the Wnt thumb were kept in the final model but with lower occupancy (0.2). To avoid overinterpretation, all sidechains were truncated to Cβ except for prolines, cysteines, and the palmitoleate (PAM)-modified serine. The PAM acyl torsion angles were also manually adjusted from the initial model. The model was refined with Phenix (53) using global minimization and B-factor with the starting model as a reference model. The blurred map with a 200 Å^2^ B-factor was used for the refinement to avoid overfitting, and the model resolution was estimated to be 9.7 Å with the model-FSC correlation cutoff of 0.5. AlphaFold2 (54) was used to model hLRP6_E1E4_ for superimposition to the XWnt8–mFzd8_CRD_–hLRP6_E1E2_ model. The structure and map were visualized using UCSF ChimeraX and PyMol (Schrödinger, LLC). The secondary structures in the figures were assigned using DSSP (55).

### Preparation of haXWnt8 NC-Linker-YW210 and Crystallization with hLRP6_E1E2_.

A fusion protein of disulfide-cyclized haXWnt8 NC-linker with YW210 Fab was designed based upon inspection of our cryo-EM structure and the previously reported structure of YW210 bound to LRP6_E1_ (25), with heterodimerization promoted using AcidP1/BaseP1 zipper fusions (56) and Crystal Kappa mutations in the light chain to promote crystallization (57). A synthesized single-chain gene containing the disulfide-cyclized haXWnt8 NC-linker (residues 227 to 243 with S228C and S234C mutations), a (GGGGS)_3_ linker, the YW210 Fab heavy chain, and 3C protease site was cloned into the pAcGP67A backbone containing a C-terminal AcidP1 leucine zipper, BAP tag, and hexahistidine tag. A synthesized gene containing the YW210 Fab light chain, including Crystal Kappa mutations to promote crystallization and 3C protease site, was cloned into the pAcGP67A backbone containing a C-terminal BaseP1 leucine zipper, BAP tag, and hexahistidine tag. P1 baculovirus was prepared as described above. The Fab chains were coexpressed in *T. ni* cells (Expression Systems) for 72 h maintained in ESF 921 Insect Cell Culture Medium (Expression Systems) at 27 °C with ambient CO_2_ and gentle agitation. Secreted protein was purified on Ni-NTA (Qiagen), digested overnight with 3C protease, and purified over a Superdex 200 10/300 GL SEC column (Cytiva) equilibrated with HBS. Purified peptide-Fab fusion was mixed with an equimolar amount of hLRP6_E1E2_, digested with carboxypeptidases A and B (Sigma) at room temperature for 1 h, and repurified by SEC. The complex was concentrated to 8 mg/mL and crystallized at 20 °C by vapor diffusion against 0.1 M succinate/phosphate/glycine buffer, pH 6.0, 25% (w/v) PEG 1500. Crystals were cryoprotected by addition of 10% (v/v) glycerol and flash cooled in liquid nitrogen.

### Crystallographic Data Collection and Refinement.

Initial diffraction data to 2.7 Å resolution were measured at Advanced Light Source beamline 2.0.1 using B4 autocollect, with a second 1.7 Å dataset measured at Stanford Synchrotron Radiation Lightsource beamline 12-1. Diffraction images were indexed, integrated and scaled using XDS, and merged using aimless (58). The structure was solved by molecular replacement in Phaser (59) using structures of hLRP6_E1E2_ (PDB 3S94) and YW210 bound to hLRP6_E1_ (PDB 3SOB) as search models. The haXWnt8 peptide was built into electron density that appeared in the molecular replacement solution. The final model was prepared by iterative cycles of rebuilding and interactive refinement in Coot (52) and reciprocal space refinement in Buster (60) and Phenix (53). The final refinement in Phenix included TLS zones chosen using TLSMD (61). Model quality was assessed using MolProbity (62). Surface area calculations were performed using PISA (63). Crystal structure figures were prepared with ChimeraX (51) and LigPlot (64). Crystallographic software was managed by SBGrid (65).

### Peptide Synthesis.

Peptide synthesis was outsourced to ChemPartner (Shanghai, China); all the linker peptides were synthesized using standard 9-fluorenylmethoxycarbonyl protocol as described earlier (66, 67). They were purified by reverse-phase high performance liquid chromatography. Peptide quality (>90% purity) was verified by liquid chromatography coupled to mass spectroscopy.

### Protein Preparation for ELISA.

Human LRP6 extracellular fragments, hLRP6_E1E2_ (residues 20 to 631) and hLRP6_E3E4_ (residues 631 to 1,253), carrying a C-terminal hexahistidine-tag were secreted from *T. ni* cells. The secreted proteins were isolated with an affinity column of immobilized anti-His-tag mAbs and eluted with buffer containing 50 mM sodium acetate pH 4.8 and 300 mM NaCl. The eluted proteins were further purified by SEC on a Superdex S200 column. The SEC column was equilibrated with buffer containing 10 mM sodium citrate pH 5.6 and 300 mM NaCl for hLRP6_E1E2_ and 10 mM sodium cacodylate pH 6.5 and 300 mM NaCl for hLRP6_E3E4_. Monomeric fractions were pooled, concentrated to ~10 mg/mL, flash-frozen, and stored at −80 °C until further usage.

LRP6-binding Fabs YW210 and YW211 were expressed as follows: 10 mL of inoculation culture was grown from *E. coli* 67A6 transformed with a Fab-encoding plasmid in Luria Broth (LB) media with 50 μg/mL carbenicillin overnight at 30 °C. The inoculation culture was added to 500 mL Soy CRAP media with 50 μg/mL carbenicillin and incubated overnight at 30 °C with shaking. Expression cultures were spun down at 10,000 *g* for 10 min. Cell pellets were resuspended in buffer containing 200 mL PBS, 25 mM EDTA, and Complete Protease Inhibitor Cocktail tablets (Roche, 1 tablet per 50 mL buffer). The mixture was homogenized and then passed twice through a microfluidizer. The suspension was centrifuged at 20,000 *g* for 45 min. The protein was loaded onto a Protein G column (Cytiva, 5 mL) equilibrated with PBS. The column was then washed with PBS and eluted with 0.6% (v/v) acetic acid. Proteins were subsequently purified by a HiTrap SP HP column (Cytiva) in buffer containing 25 mM MES pH 5.5 and eluted with a linear gradient of buffer containing 25 mM MES pH 5.5 and 500 mM NaCl. Monomeric fractions were pooled, concentrated to ~150 μM, flash-frozen, and stored at −80 °C until further usage. H07 VHH was provided by the Genentech Biomolecular Resources.

### ELISA.

ELISA was carried out on Nunc Maxisorp plates (Thermo Scientific) coated with 5 μg/mL neutravidin. The blocking was performed with 0.5% (w/v) BSA in PBS for 2 h at room temperature, and 1 μg of biotinylated linker peptide per well was immobilized. Serial dilutions of hexahistidine-tagged LRP6 domains or Reck (R&D systems) were prepared in ELISA buffer [PBS, 0.5% (w/v) BSA, and 0.05% (v/v) tween 20] and added to the wells. The reaction mixture was incubated for 45 min at room temperature and washed with ELISA buffer. Horseradish peroxidase-conjugated anti-His antibody (Abcam, ab1187) was diluted in ELISA buffer at 1:5,000 and added to the wells for 45 min incubation. Linker peptide-bound LRP6 domains or Reck was detected using TMB peroxidase substrate (Seracare, 2-component system), and the plates were read at 450 nm using SpectraMax M5e (Molecular Devices). For the competition assays with Fab YW210, Fab YW211, or H07 VHH, a constant concentration of LRP6 domains at 3x EC50 (*SI Appendix*, Table S3) was used. A serial dilution of competitors was added, and the bound His-tagged LRP6 domains were detected as described.

### TOPbrite Dual-Luciferase Wnt Reporter Assays.

HEK293 cells with stably integrated firefly-luciferase-based Wnt reporter (TOPbrite) (66) and pRL-SV40 Renilla luciferase (Promega) were maintained in a 5% CO_2_ humidified incubator at 37 °C in DMEM with nutrient mixture F12 (50:50), 10% (v/v) FBS, 2 mM GlutaMAX (Gibco), and 40 μg/mL hygromycin (Cellgro). Cells were grown for at least 24 h before any experiments. 20,000 to 40,000 cells/well in 50 μL medium were seeded in each well of clear-bottom white polystyrene 96-well plates (Falcon) and incubated for 24 h. The cells were then transfected with 0 to 25 ng of Wnt or Wnt chimera-expressing constructs (Wnt3a_1, Wnt5a_1, and Wnt3a_haXWnt8) mixed with FuGENE HD in 10 μL OptiMEM, followed by incubation for 24 to 48 h. Treatment with peptides or LRP6 Fabs was done for 6 h before the assay measurement. Readout was obtained with 50 μL of Dual-Glo Luciferase Assay system (Promega) according to the manufacturer’s instructions on a Perkin Elmer EnVision multilabel reader. The ratios of firefly luminescence to Renilla luminescence were calculated, and in some instances normalized to control (nontreated) samples. Cell lines were tested for *mycoplasma* contamination and authenticated by single-nucleotide polymorphism analysis.

### Western Blot Analysis.

For Wnt expression assay, 4.0 × 10^6^ HEK293 luciferase reporter cells were seeded onto 10-cm dishes and transfected on the following day with 4 μg DNA encoding Wnt1, Wnt3a, Wnt5a, Wnt1_3a, Wnt3a_1, and Wnt5a_1 using FuGENE HD. Empty vector (pcDNA3.1) was used as control. After 48 h of incubation, culture medium was collected and cells were gently washed twice with 5 mL cold PBS and lysed in 0.5 mL of lysis buffer [PBS, 1% (v/v) Triton X-100 and protease inhibitor cocktail (Roche)]. The collected lysate was centrifuged for 5 min at 18,400 *g* to remove cell debris, and the supernatant was used to determine total protein concentration by BCA assay (Thermo Fisher Scientific). 10 μg of the total protein was loaded onto an SDS-PAGE (4 to 12%) to assess Wnt protein levels. To evaluate secreted Wnt protein levels, the collected Wnt-conditioned medium (6 mL) was incubated with 50 μL Blue Sepharose beads (Cytiva) on a rocking platform overnight at 4 °C. The beads were washed three times with 500 μL PBS containing 0.1% (v/v) Tween 20 by centrifugation at 2,400 *g* for 5 min. 50 μL of 1× XT sample buffer including reducing reagent (Bio-rad) was added to the beads and the sample was boiled for 5 min at 95 °C before loading onto an SDS-PAGE (4 to 12%). Proteins were then transferred to nitrocellulose membrane and probed with antibodies against Wnt1 (Abcam), Wnt3a (Cell Signaling), Wnt 5a/b (Cell signaling), and HSP90 (Cell Signaling).

For the measurement of LRP6/β-catenin levels, 2.0 × 10^5^ HEK293 luciferase reporter cells were seeded onto 24-well plates and transfected next day with 250 ng of DNA encoding Wnt proteins and chimeras as described above using FuGENE HD. After 24 h incubation in regular condition, cells were gently washed twice with 500 μL cold PBS and directly lysed in 50 μL of lysis buffer [PBS, 1% (v/v) Triton X-100 and protease inhibitor cocktail (Roche)]. The collected lysate was centrifuged for 5 min at 18,400 *g* to remove cell debris and the supernatant was used to determine total protein concentration by BCA assay. 15 μg of total protein was loaded onto an SDS-PAGE (4 to 12%) to assess LRP6 and β-catenin protein levels using phospho- and total LRP6 (Cell Signaling), active- and total β-catenin (Cell Signaling), and GAPDH (Cell Signaling) antibodies.

Antibodies were purchased from Abcam [Wnt1: ab15251 (Lot number GR3315807-3, dilution 1:1,000)] and Cell Signaling Technology [Wnt3a: 2391S (Lot number 2, dilution 1:1,000), Wnt5a/b (C27E8): 2530T (Lot number 4, dilution 1:1,000), HSP90 (C45G5): 4877S (Lot number 5, dilution 1:2,000), GAPDH (14C10): 2118S (Lot number 14, dilution 1:2,000), LRP6 (C4C7): 2560S (Lot number 11, dilution 1:1,000), Phospho-LRP6 (Ser1490): 2568S (Lot number 6, dilution 1:500), β-catenin (D10A8): 8480S (Lot number 5, 1:1,000) and Non-phospho (active) β -catenin (Ser33/37/Thr41) (D13A1): 8814S (Lot number 7, 1:500)].

### Quantitative RT-PCR Analysis.

4.0 × 10^5^ HEK293 cells were seeded onto 12-well plates and transfected next day with 500 ng of DNA encoding Wnt proteins and chimeras, Wnt1, Wnt3a, Wnt5a, Wnt1_3a, Wnt3a_1, and Wnt5a_1, using FuGENE HD. After 24 h incubation, cells were gently washed twice with 500 μL cold PBS and RNA was isolated using the RNeasy kit (Qiagen), as instructed by the manufacturer’s manual. Briefly, cells were lysed in RLT buffer including β-mercaptoethanol, which was followed by addition of 70% ethanol. The sample was then transferred to the column provided in the kit. After several washing steps, RNA was eluted using RNAse-free water. The real-time PCR reactions were performed with the TaqMan RNA-to-C_T_ 1-Step Kit (Applied Biosystems) by preparing samples as follows (total 10 μL reaction): 5.0 μL of 2X TaqMan RT-PCR mix, 0.5 μL of 20X TaqMan gene expression probe, 0.25 μL of 40X TaqMan RT enzyme mix, 50 ng of RNA (2 μL, 25 ng/μL), and 2.25 μL of nuclease-free water. The reaction was initiated at 48 °C for reverse transcription for 15 min, followed by 45 amplification cycles (activation of AmpliTaq Gold DNA polymerase at 95 °C for 10 min, denaturation at 95 °C for 15 s, and anneal/extend at 95 °C for 1 min). The assay was run on QuanStudio 7 Flex Real-Time PCR systems (Thermo Fisher Scientific). Relative RNA levels were calculated using the ΔΔC*_T_* method and normalized to the level of housekeeping gene human HRPT1 within the same sample and further normalized to the sample from cells transfected with control empty vector. Taqman RNA-to CT 1 step kit (4392653) and gene expression probes (Axin2-FAM, Hs01063168-m1 and HPRT1-FAM, Hs02800695_m1) were purchased from Thermo Fisher Scientific.

## Supplementary Material

Appendix 01 (PDF)

## Data Availability

The cryo-EM map has been deposited in the Electron Microscopy Data Bank under accession code EMD-26989 (68), and the corresponding model coordinate has been deposited to Protein Data Bank (PDB) under accession number 8CTG (69). The crystal structure has been deposited to PDB under accession number 8FFE (70), and diffraction images were deposited in the SBGrid Data Bank (71, 72).
