# Supplementary material for: Structure of the Wnt–Frizzled–LRP6 initiation complex reveals the basis for coreceptor discrimination
Source: Proc Natl Acad Sci U S A. 2023 Mar 9;120(11):e2218238120. doi: 10.1073/pnas.2218238120 (PMC10089208; doi:10.1073/pnas.2218238120)
Supplement: Supplementary file 1 — Appendix 01 (PDF) [file pnas.2218238120.sapp.pdf]

PNAS

Supporting Information for
Structure of the Wnt–Frizzled–LRP6 initiation complex reveals the basis for coreceptor discrimination

Naotaka Tsutsumi†, Sunhee Hwang†, Deepa Waghray†, Simon Hansen†, Kevin M. Jude, Nan Wang, Yi Miao, Caleb R. Glassman, Nathanael A. Caveney, Claudia Y. Janda, Rami N. Hannoush*, and K. Christopher Garcia*

Rami N. Hannoush* and K. Christopher Garcia*
Email: hannoush.rami@gene.com, kcgarcia@stanford.edu

This PDF file includes:

Figures S1 to S11
Tables S1 to S3
Appendix 1

A

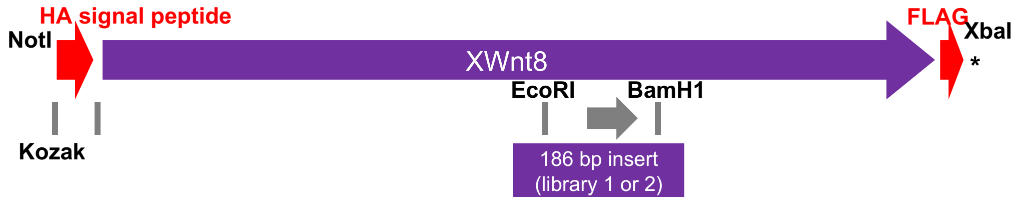

				library 1		library 2	
hWnt8a		XWnt8		codon	possible amino acids	codon	possible amino acids
E	219	E	220				
M	220	M	221				
D	221	D	222				
K	222	K	223	MRK	3xR, K, H, N, Q, S		
R	223	R	224	MRK	3xR, K, H, N, Q, S		
Q	224	K	225	MRK	3xR, K, H, N, Q, S		
L	225	M	226	VWG	K, Q, E, L, M, V	VWG	K, Q, E, L, M, V
R	226	R	227	VST	R, T, S, A, G, P	VST	R, T, S, A, G, P
A	227	S	228			VST	R, T, S, A, G, P
G	228	G	229			RVT	N, D, T, S, A, G
N	229	N	230	MRT	H, N, S, R	MRT	H, N, S, R
S	230	S	231			RVT	N, D, T, S, A, G
A	231	A	232			KYG	S, A, L, V
E	232	D	233	VAW	K, H, N, Q, D, E	VAW	K, H, N, Q, D, E
G	233	N	234			RVT	N, D, T, S, A, G
H	234	R	235	VDT	R, H, N, S, G, D, I, L V		
W	235	G	236	BGG	R, G, W	DSG	R, T, S, A, G, W
V	236	A	237				
P	237	I	238				
A	238	A	239				
E	239	D	240				
A	240	A	241				
F	241	F	242				

B

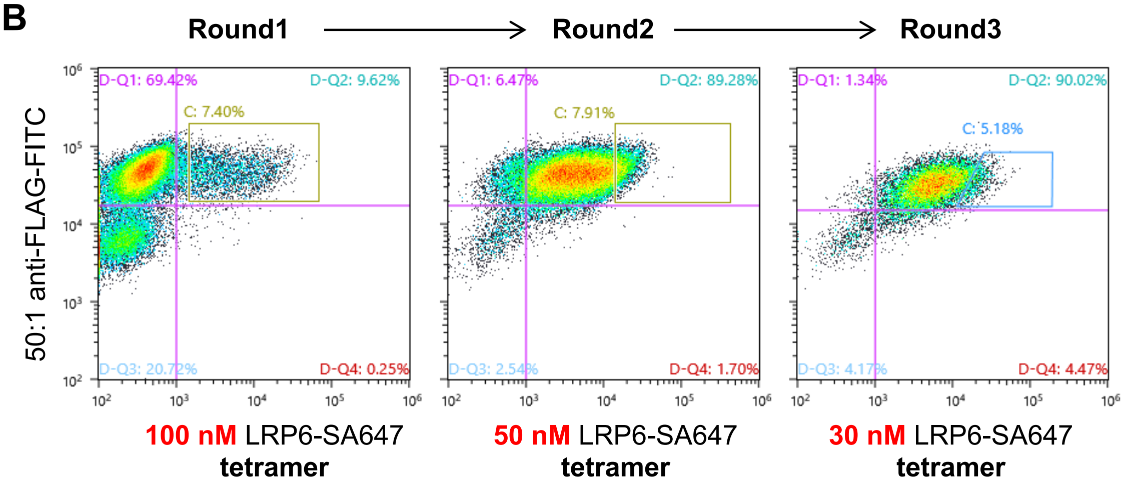

Fig. S1. Construction and selection of the XWnt8 NC-linker library
(A) Design of the XWnt8 NC-linker libraries. (B) FACS plot and gating during the round 1, 2, and 3 selections for the XWnt8 NC-linker library displayed on the mammalian cell surface.

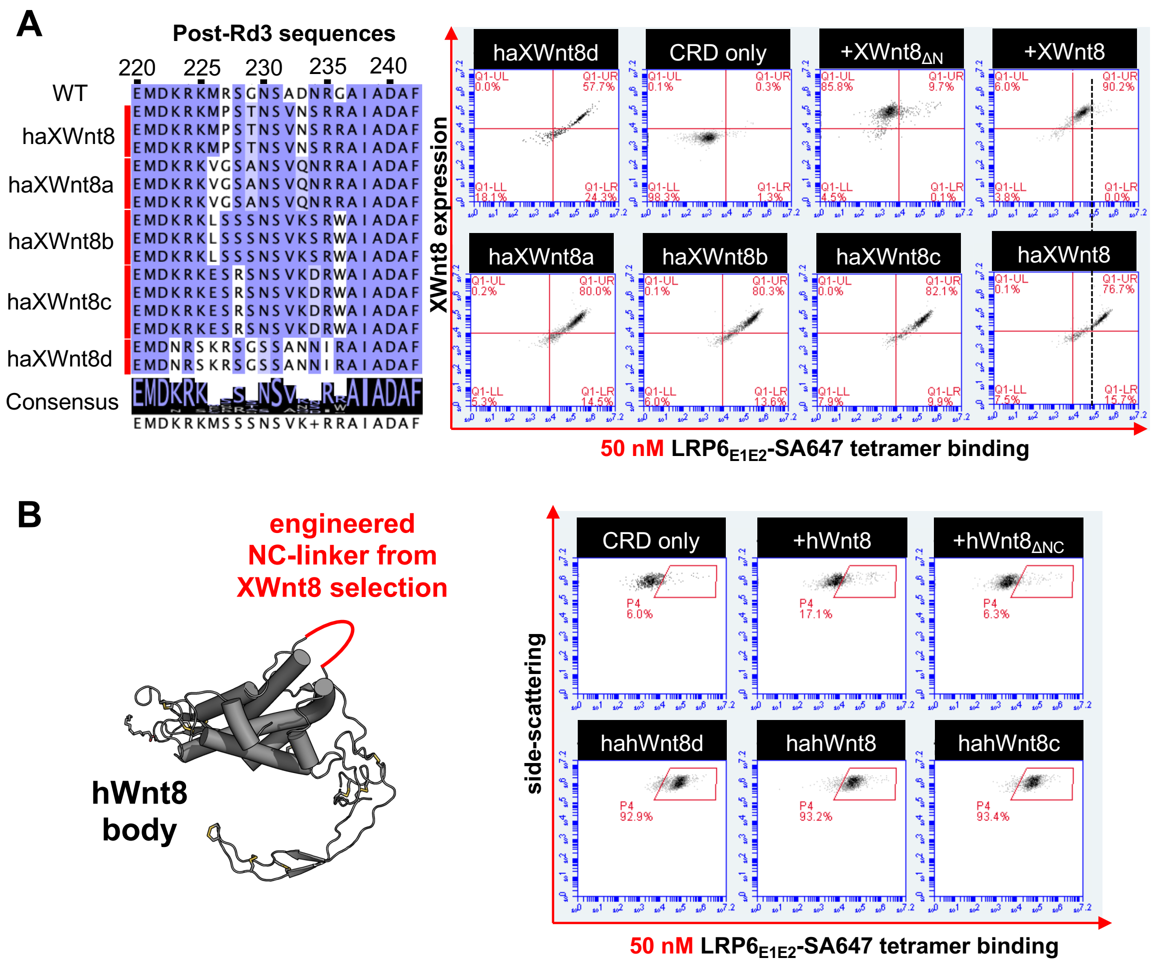

Fig. S2. Single clones from the post-round 3 XWnt8 NC-linker library
(A) The NC-linker sequences for the five single clones after the round 3 selection in comparison with the wild-type sequence (left), and their hLRP6$_{E1E2}$ tetramer binding on the engineered cells (right). (B) The schematic of the NC-linker grafting experiment from haXWnt8s to hWnt8 (left), and the chimeras' hLRP6$_{E1E2}$ tetramer binding on the engineered cells (right).

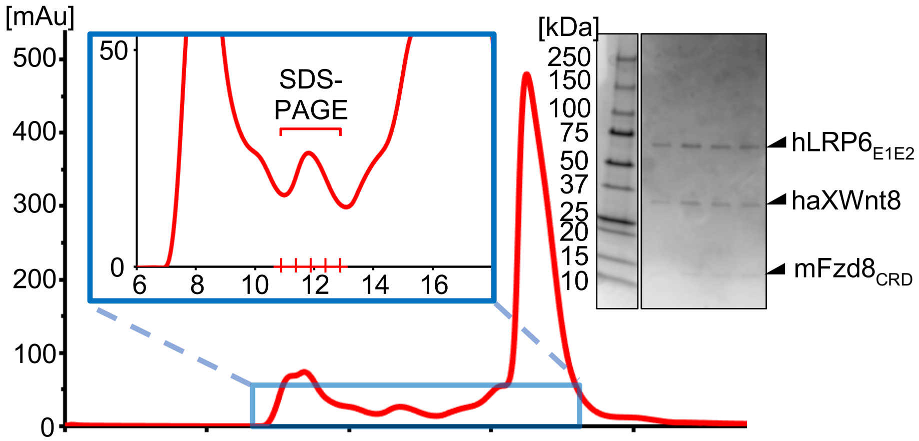

Fig. S3. Reconstitution of haXWnt8-mFzd8$_{CRD}$-hLRP6$_{E1E2}$
Size-exclusion chromatography profile and SDS-PAGE of haXWnt8-mFzd8$_{CRD}$-hLRP6$_{E1E2}$.

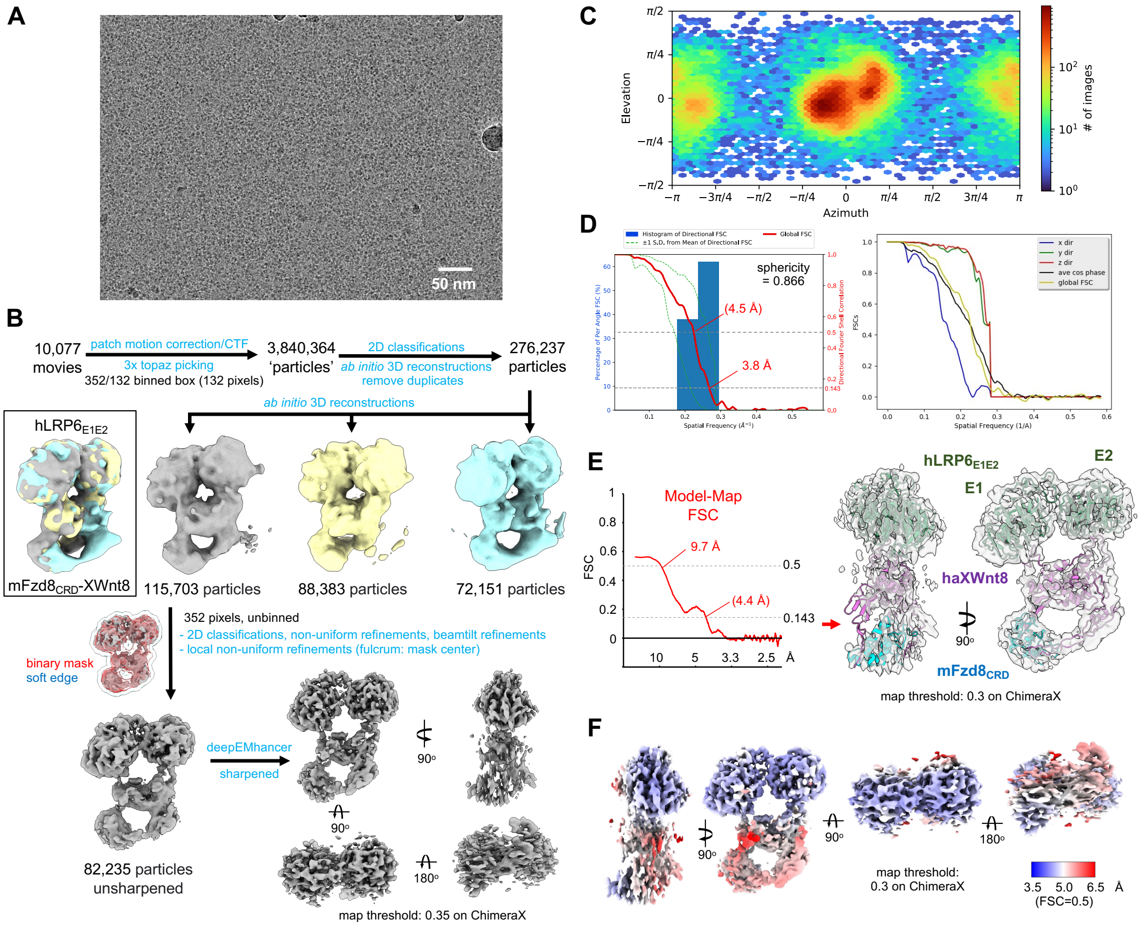

Fig. S4. Cryo-EM data analysis

(A) Representative micrograph from cryo-EM data collection. (B) Cryo-EM data analysis workflow with representative 3D reconstructions during the data processing. (C) Angular distribution of the aligned particles in the final 3D reconstruction output from cryoSPARC. (D) Overall and directional gold-standard FSC curves calculated using 3DFSC. (E) The model-map FSC curve made with auto-masking by Phenix (left), and the cryo-EM map overlayed on the model (right). The red arrow on the right panel indicates the region with possible exposure to the air-water interface. (F) Local resolution estimates by Phenix colored on the surface representation of the deepEMhancer sharpened map. Blue: higher resolution, red: lower resolution.

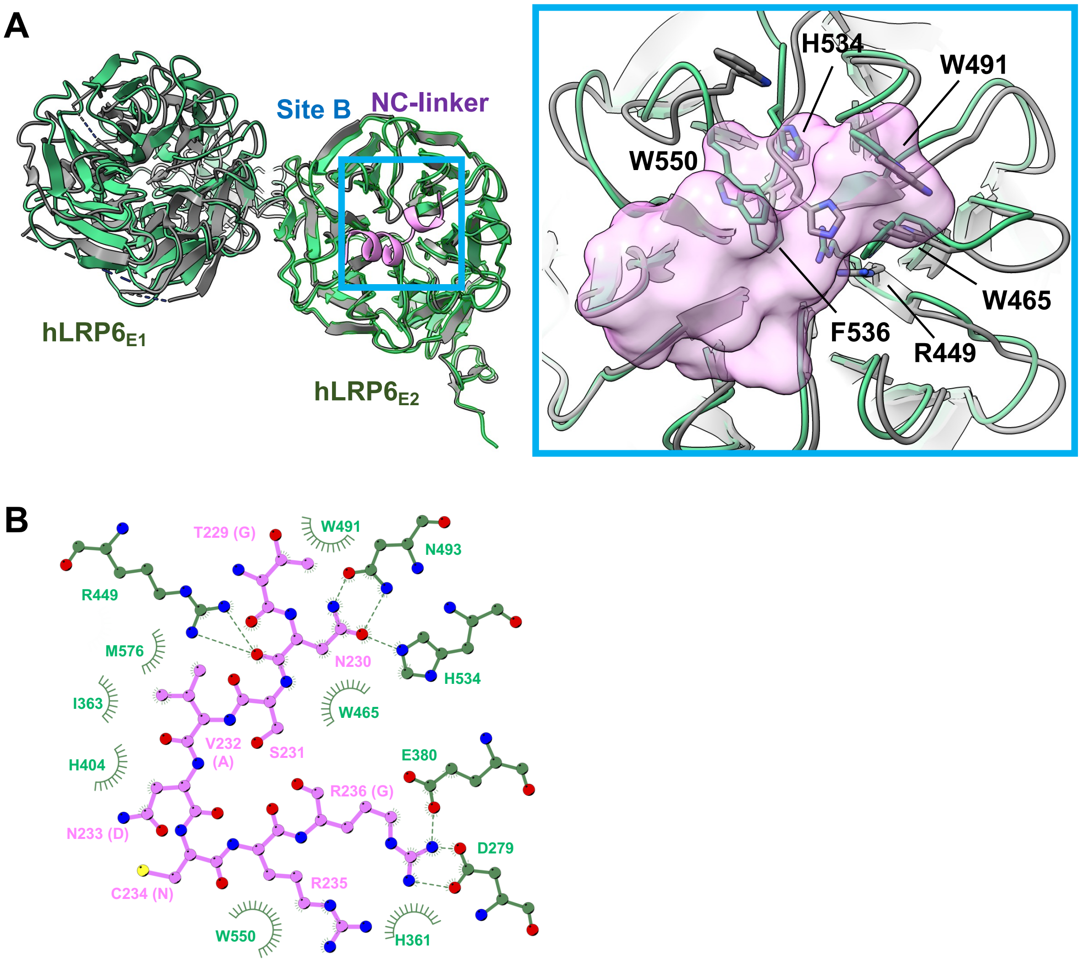

Fig. S5. Structural comparison between hLRP6$_{E1E2}$ with or without the haXWnt8 NC-linker peptide and 2D interaction plot of haXWnt8 NC-linker

(A) The crystal structure of *apo* hLRP6$_{E1E2}$ (PDB 3S94, gray) was aligned to the E2 domain of the hLRP6$_{E1E2}$ (green) bound to the NC-linker peptide (pink). The E2 funnel is zoomed in (blue box) with haXWnt8 NC-linker shown in transparent surface representation. Key residues rearranged for NC-linker binding are shown as sticks. Substantial structural rearrangement in the hLRP6$_{E2}$ funnel is required to accommodate the NC-linker peptide. In the previously published structure of the *apo* hLRP6$_{E1E2}$ (PDB 3S94, gray), H534^{LRP6} occupies the position filled by N230 at the center of the NC-linker, while F536 is buried toward the β-propeller core. Rearrangement of this loop moves H534^{LRP6} into position optimal to form the hydrogen bonding to the N230 sidechain, flips I535^{LRP6} into the domain core, and flips F536^{LRP6} out to form the V232 binding pocket. This loop, between blades 5 and 6 of the E2 β-propeller domain, is shorter in the E1, E3, or E4 β-propellers of LRP6, suggesting its importance in Wnt subtype discrimination. This movement also allows W550^{LRP6} to swing inward to the V232 pocket, with smaller movements of W491^{LRP6}, W491^{LRP6}, and R449^{LRP6} completing the formation of the binding site.
(B) A 2D interaction plot between the haXWnt8 NC-linker and LRP6. Hydrogen bonds are shown as dashed lines and van der Waals contacts as rays. For mutated residues in haXWnt8, the wild-type identity is indicated in parentheses.

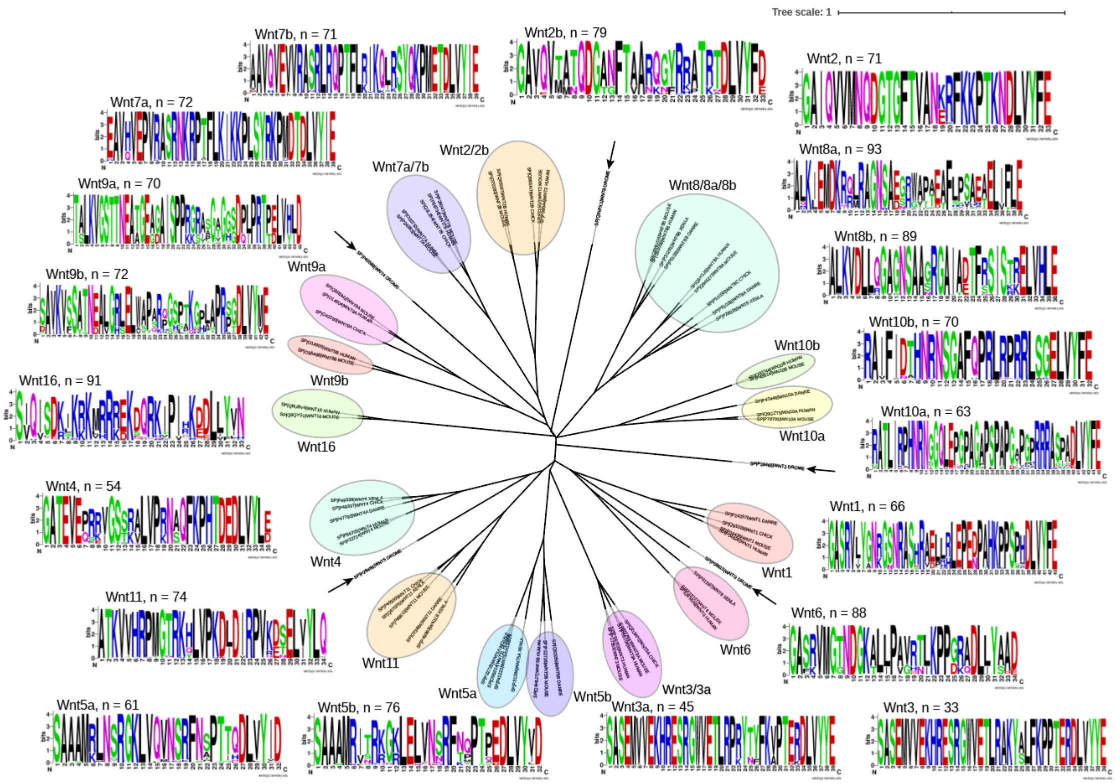

Fig. S6. Phylogenetic analysis reveals evolutionary conservation of the NC-linker

The sequences of the NC-linker of Wnts from *Homo sapiens* (humans), *Mus musculus* (mouse), *Gallus gallus* (chicken), *Danio rerio* (Zebrafish), *Xenopus laevis* (African clawed frog) and *Drosophila melanogaster* (Fruit fly) were aligned and represented as phylogenetic tree. Arrows indicate *D. melanogaster* sequences. Weblogos are shown for the NC-linker sequence of each paralog from several species (number is indicated).

Name	Linker domain sequence
Wnt1	G A S R V L Y G N R G S N R A S R A E L L R L E P E D P A H K P P S P H D L V
65	G A S R V L A G A R G S N R A S R A E L L R L E P E D P A H K P P S P H D L V
66	G A S R V L Y G N A G A N R A S R A E L L R L E P E D P A H K P P S P H D L V
67	G A S R V L Y G N R G S A A A S R A E L L R L E P E D P A H K P P S P H D L V
68	G A S R V L Y G N R G S N R A A A A E L L R L E P E D P A H K P P S P H D L V
69	G A S R V L Y G N R G S N R A S R A A A L R L E P E D P A H K P P S P H D L V
70	G A S R V L Y G N R G S N R A S R A E L A A L E P E D P A H K P P S P H D L V
71	G A S R V L Y G N R G S N R A S R A E L L R A A P E D P A H K P P S P H D L V
72	G A S R V L Y G N R G S N R A S R A E L L R L E A A D P A H K P P S P H D L V
73	G A S R V L Y G N R G S N R A S R A E L L R L E P E A A A H K P P S P H D L V
74	G A S R V L Y G N R G S N R A S R A E L L R L E P E D P A A A P P S P H D L V
75	G A S R V L Y G N R G S N R A S R A E L L R L E P E D P A H K A P A P H D L V
76	G A S R V L Y G N R G S N R A S R A E L L R L E P E D P A H K P A A P H D L V
77	G A S R V L Y G N R G S N R A S R A E L L R L E P E D P A H K P P S A A D L V

Fig. S7. Sequences of the Ala mutants in Wnt1 NC-linker
Two residues were substituted for Ala in each mutant.

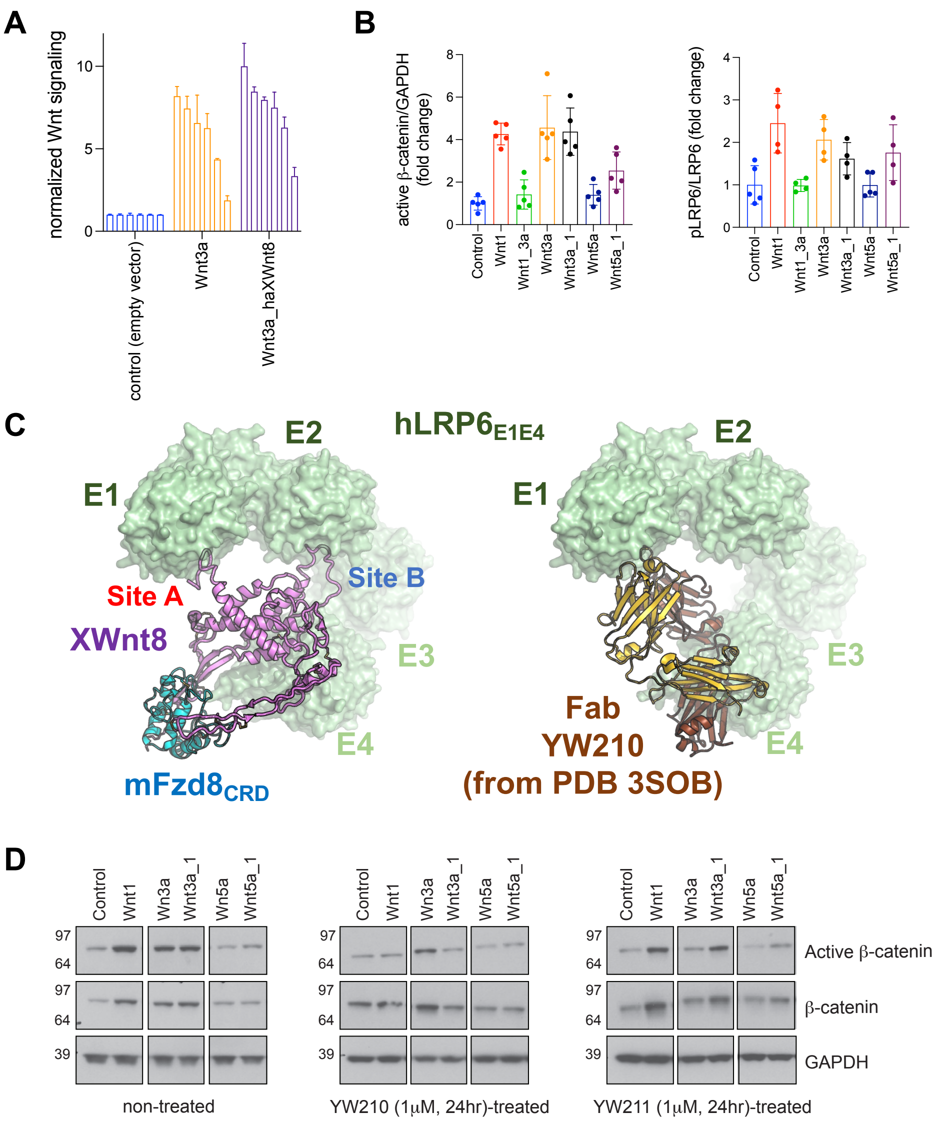

Fig. S8. LRP6 module specificity of the Wnt chimeras

(A) Representative TOPbrite dual-luciferase reporter assay showing the comparable activity of Wnt3a_haXWnt8 chimera to the activity of Wnt3a. (B) Quantification of Western blots in Figure 4C. Bar and error bar represent the mean and SD of four to five biological replicates. (C) Mechanism of Wnt antagonism by the YW210 antibody. Fab YW210 competes with the N-terminal loop of the E1E2-biding Wnts at site A (the E1 interface), and sterically crashes with Wnt's globular body, thereby inhibiting the site B-driven signaling complex formation. On the right panel, the crystal structure of hLRP6$_{E1}$-YW210 Fab (PDB 3SOB) is superimposed onto the hLRP6$_{E1E4}$ AlphaFold2 model to compare with the left panel which is identical to Figure 6B left. (D) Representative Western blots showing β-catenin and LRP6 levels with overexpression of Wnts and Wnt chimeras in the presence of 1 μM Fab YW210 and Fab YW211. The treatment by Fab YW210 significantly attenuated active β-catenin levels compared to the control, whereas Fab YW211 only affected Wnt3a signaling. Representative Western blot from one of three biological replicates.

A

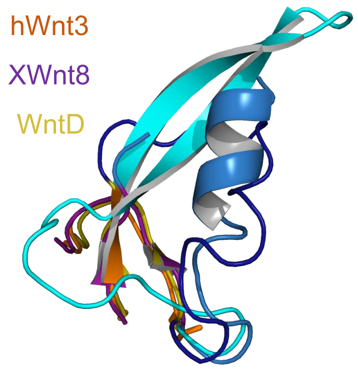

B

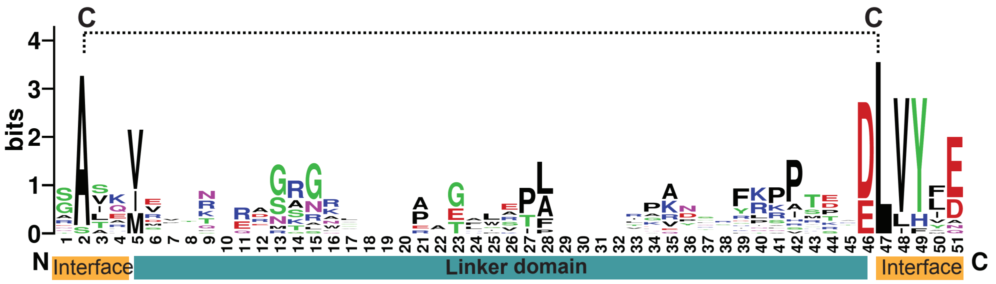

Fig. S9. Sequence diversity of the NC-linker of Wnt isoforms
(A) Close-up view of the NC-linker. Superposition of all three available NC-linker in shades of blue and interface motifs in orange, purple and yellow (PDB hWnt3; 6AHY, XWnt8; 4F0A and WntD; 4KRR). (B) Weblogo shows the sequence conservation of the interface motifs and diversity of the NC-linker of all 19 human Wnts. Introduced disulfide bridge is indicated by black dashed line.

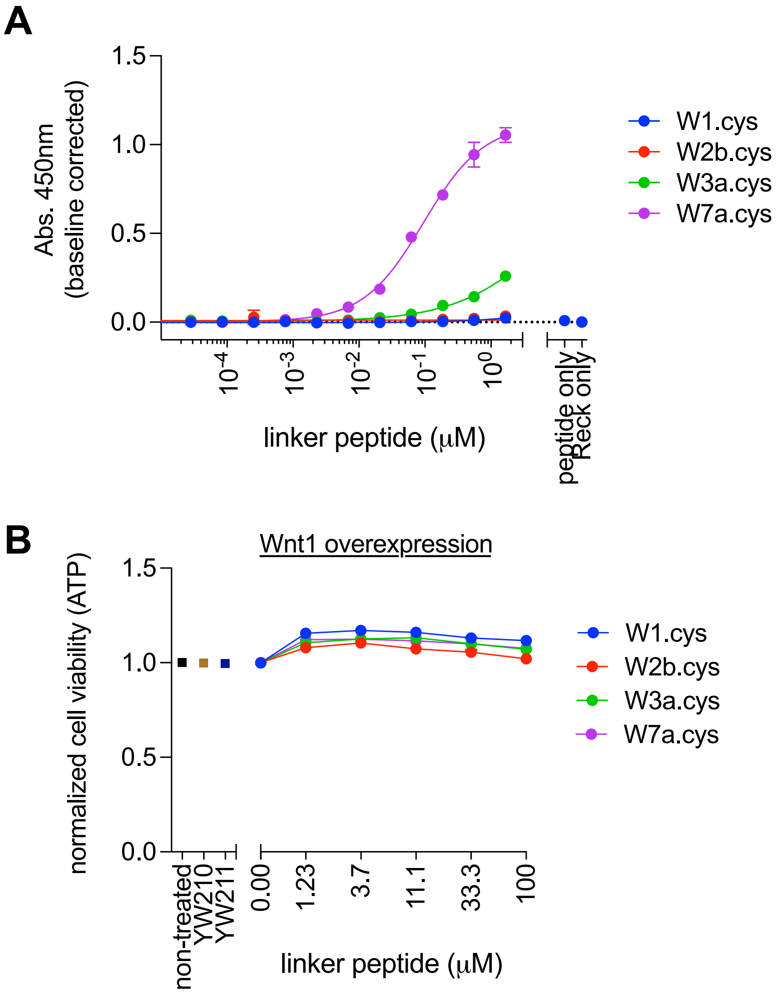

Fig. S10. Specificity of W7a.cys peptide and effect of NC-linker peptides on cell viability

(A) ELISA showing the specific binding of W7a.cys peptide to co-receptor Reck. (B) The linker peptides did not cause toxicity to cells, indicating that the inhibitory effect of W1.cys peptide on Wnt1-mediated signaling (Figure 4D) was induced by the peptide. Bar and error bar represent the mean and SD of five technical replicates.

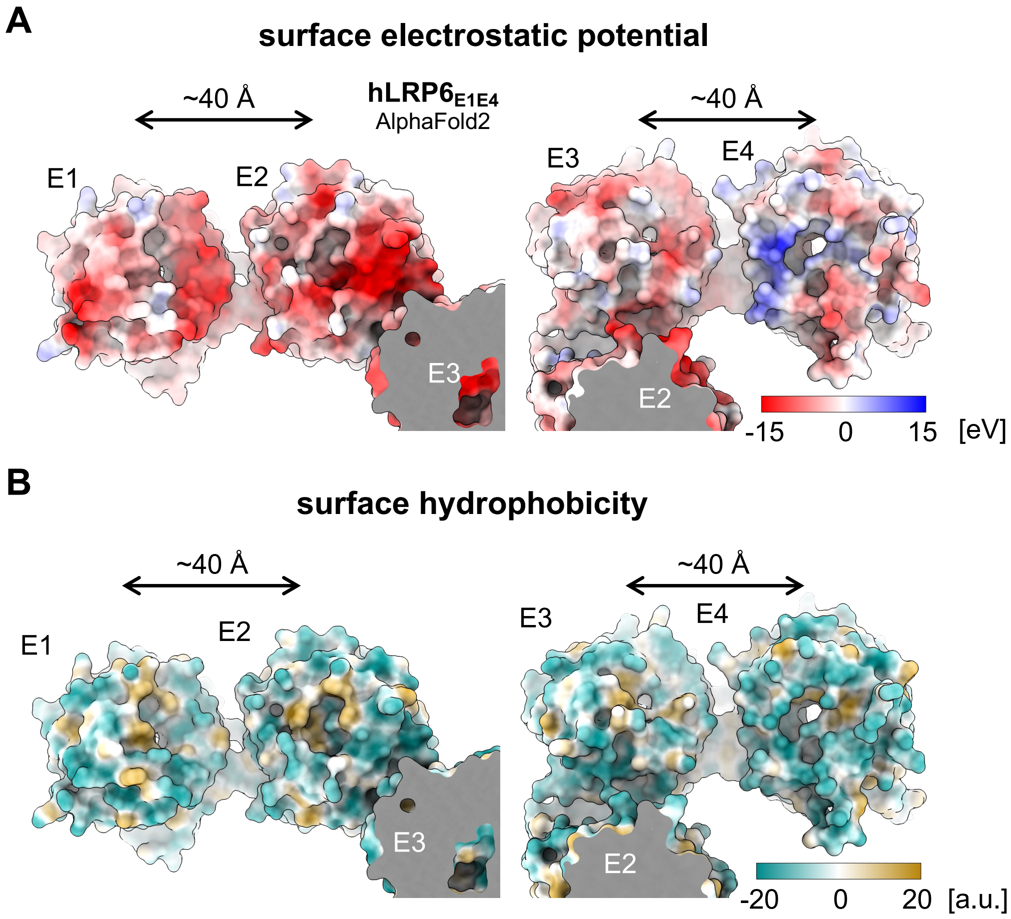

Fig. S11. Modularity of $LRP6_{E1E2}$ and $LRP6_{E3E4}$

(A) Electrostatic potentials of $hLRP6_{E1E4}$ looking down the β-propeller funnels.

(B) Hydrophobicity of LRP6 funnels. The AlphaFold2 $hLRP6_{E1E4}$ model in surface representation were colored as indicated in the panel, and the figure is prepared with UCSF ChimeraX. Red, more acidic; Blue, more basic; Brown, more hydrophobic; Green, more hydrophilic.

Table S1. Cryo-EM data collection and modeling

	haXWnt8-mFzd8$_{CRD}$-hLRP6$_{E1E2}$ (EMDB EMD-26989, PDB 8CTG)
**Data collection and processing**	
Nominal magnification	29,000x
Calibrated magnification	58,680x
Voltage (kV)	300
Electron exposure (e/Å^2)	55
Defocus range (μm)	-1.0 to -2.0
Pixel size (Å)	0.8521
Symmetry imposed	C2
Initial “particle” images (no.)	3,840,364
Final particle images (no.)	82,235
Map resolution (Å)	3.8 (4.5)
FSC threshold	0.143 (0.5)
Map resolution range (Å)	4.4 to 7.2
FSC threshold	0.5
**Refinement**	
Initial models used (PDB codes)	4F0A and 3S94
Model resolution (Å)	9.7 (4.4)
FSC threshold	0.5 (0.143)
Map sharpening method	deepEMhanceer (visualization)<br>uniform B-factor of 200 Å^2<br>(blurred, refinement)
Model composition	
Non-hydrogen atoms	5,100
Protein residues	1,006
Ligands	PAM
B factors (Å^2)	
Protein	267.4
Ligand	551.1 (PAM)
R.m.s. deviations	
Bond lengths (Å)	0.004
Bond angles (°)	0.975
Validation	
MolProbity score	2.83
Clashscore	12.46
Poor rotamers (%)	6.90
Ramachandran plot	
Favored (%)	88.53
Allowed (%)	9.66
Disallowed (%)	1.81

Table S2. X-ray crystallographic data collection and refinement statistics

	haXWnt8 NC-linker-YW210 Fab-hLRP6$_{E1E2}$ (PDB 8FFE)
**Data collection and processing**	
Number of Crystals	1
Wavelength (Å)	0.9795
Resolution range (Å)	44.37 - 1.72 (1.781 - 1.72)
Space group	*C2*
Cell dimensions	
a, b, c (Å)	125.212 91.385 104.055
α, β, γ (°)	90 103.667 90
Total reflections	777534 (44840)
Unique reflections	109415 (6719)
Multiplicity	7.1 (6.7)
Completeness (%)	90.56 (55.47)
Mean $I / \sigma I$	10.49 (0.64)
Wilson B-factor	30.74
R_{merge}	0.09658 (2.068)
R_{meas}	0.1042 (2.243)
R_{pim}	0.03889 (0.8556)
*CC*1/2	0.997 (0.426)
**Refinement**	
Reflections used in refinement	109300 (6623)
Reflections used for R_{free}	5465 (328)
R_{work}	0.1853 (0.4209)
R_{free}	0.2313 (0.4618)
Number of non-hydrogen atoms	
macromolecules	8324
ligands	199
solvent	593
Protein residues	1067
R.m.s. deviations (bonds, Å)	0.009
R.m.s. deviations (angles, °)	0.98
Ramachandran favored (%)	95.36
Ramachandran allowed (%)	4.54
Ramachandran outliers (%)	0.09
Rotamer outliers (%)	1.86
Clashscore	3.52
Average B-factor	38.70
macromolecules	38.10
ligands	67.11
solvent	37.61
Number of TLS groups	14

Statistics for the highest-resolution shell are shown in parentheses.

Table S3. EC_{50} and R_{max} values determined by ELISA

Peptide	EC_{50} (μM), E1E2	R_{max} (a.u.), E1E2	EC_{50} (μM), E3E4	R_{max} (a.u.), E3E4
W1.cys	0.067 ± 0.047	2.22 ± 0.31	-	
W2b.cys	0.018 ± 0.015	2.06 ± 0.24	-	
W3a.cys	0.22 ± 0.092	0.99 ± 0.53	0.19 ± 0.16	1.47± 0.29
W7a.cys	0.04 ± 0.018	2.13 ± 0.09	-	

Appendix 1 Protein sequences used for the signaling assays with DNA transfection

Wild types:

Linker domain colored in green

hWnt1:

>sp|P04628|WNT1_HUMAN Proto-oncogene Wnt-1 OS=Homo sapiens OX=9606 GN=WNT1 PE=1 SV=1
MGLWALLPGWVSATLLLALAALPAALAANSSGRWWGIVNVASSTNLLTDSKSLQLVLEPSLQLLSRKQRRL
IRQNPGILHSVSGGLQSAVRECKWQFRNRRWNCPTAPGPHLFGKIVNRGCRETAFIFAITSAGVTHSVARS
CSEGSIESCTCDYRRRGPGGPDWHWGGCSDNIDFGRLFGREFVDSGEKGRDLRFLMNLHNNEAGRTTVFSE
MRQECKCHGMSGSCTVRTCWMRLPTLRAVGDVLRDRFDGASRVLYGNRGSNRASRAELLRLEPEDPAHKPP
SPHDLVYFEKSPNFCTYSGRLGTAGTAGRACNSSSPALDGCELLCCGRGHRTRTQRVTERCNCTFHWCCHV
SCRNCTHTRVLHECL

hWnt3a:

>sp|P56704|WNT3A_HUMAN Protein Wnt-3a OS=Homo sapiens OX=9606 GN=WNT3A PE=1 SV=2
MAPLGYFLLLCSLKQALGSYPIWWSLAVGPQYSSLGSQPILCASIPGLVPKQLRFCRNYVEIMPSVAEGIK
IGIQECQHQFRGRRWNCTTVHDSLAIFGPVLDKATRESAFVHAIASAGVAFAVTRSCAEGTAAICGCSSRH
QGSPGKGWKWGGCSEDIEFGGMVSREFADARENRPDARSAMNRHNNEAGRQAIASHMHLKCKCHGLSGSCE
VKTCWWSQPDFRAIGDFLKDKYDSASEMVVEKHRESRGWVETLRPRYTYFKVPTERDLVYYEASPNFCEPN
PETGSFGTRDRTCNVSSHGIDGCDLLCCGRGHNARAERRREKCRCVFHWCCYVSCQECTRVYDVHTCK

hWnt5a:

>sp|P41221|WNT5A_HUMAN Protein Wnt-5a OS=Homo sapiens OX=9606 GN=WNT5A PE=1 SV=2
MKKSIGILSPGVALGMAGSAMSSKFFLVALAIFFSFAQVVIEANSWWSLGMNNPVQMSEVYIIGAQPLCSQ
LAGLSQGQKKLCHLYQDHMQYIGEGAKTGIKECQYQFRHRRWNCSTVDNTSVFGRVMQIGSRETAFTYAVS
AAGVVNAMSRACREGELSTCGCSRAARPKDLPRDWLWGGCGDNIDYGYRFAKEFVDARERERIHAKGSYES
ARILMNLHNNEAGRRTVYNLADVACKCHGVSGSCSLKTCWLQLADFRKVGDALKEKYDSAAAMRLNSRGKL
VQVNSRFNSPTTQDLVYIDPSPDYCVRNESTGSLGTQGRLCNKTSEGMDGCELMCCGRGYDQFKTVQTERC
HCKFHWCCYVKCKKCTEIVDQFVCK

Chimeras:

Wnt1_3a:

MGLWALLPGWVSATLLLALAALPAALAANSSGRWWGIVNVASSTNLLTDSKSLQLVLEPSLQLLSRKQRRL
IRQNPGILHSVSGGLQSAVRECKWQFRNRRWNCPTAPGPHLFGKIVNRGCRETAFIFAITSAGVTHSVARS
CSEGSIESCTCDYRRRGPGGPDWHWGGCSDNIDFGRLFGREFVDSGEKGRDLRFLMNLHNNEAGRTTVFSE
MRQECKCHGMSGSCTVRTCWMRLPTLRAVGDVLRDRFDSASEMVVEKHRESRGWVETLRPRYTYFKVPTER
DLVYFEKSPNFCTYSGRLGTAGTAGRACNSSSPALDGCELLCCGRGHRTRTQRVTERCNCTFHWCCHVSCR
NCTHTRVLHECL

Wnt3a_1:

MAPLGYFLLLCSLKQALGSYPIWWSLAVGPQYSSLGSQPILCASIPGLVPKQLRFCRNYVEIMPSVAEGIK
IGIQECQHQFRGRRWNCTTVHDSLAIFGPVLDKATRESAFVHAIASAGVAFAVTRSCAEGTAAICGCSSRH
QGSPGKGWKWGGCSEDIEFGGMVSREFADARENRPDARSAMNRHNNEAGRQAIASHMHLKCKCHGLSGSCE
VKTCWWSQPDFRAIGDFLKDKYDGASRVLYGNRGSNRASRAELLRLEPEDPAHKPPSPHDLVYYEASPNFC
EPNPETGSFGTRDRTCNVSSHGIDGCDLLCCGRGHNARAERRREKCRCVFHWCCYVSCQECTRVYDVHTCK

Wnt5a_1:

MKKSIGILSPGVALGMAGSAMSSKFFLVALAIFFSFAQVVIEANSWWSLGMNNPVQMSEVYIIGAQPLCSQ
LAGLSQGQKKLCHLYQDHMQYIGEGAKTGIKECQYQFRHRRWNCSTVDNTSVFGRVMQIGSRETAFTYAVS
AAGVVNAMSRACREGELSTCGCSRAARPKDLPRDWLWGGCGDNIDYGYRFAKEFVDARERERIHAKGSYES
ARILMNLHNNEAGRRTVYNLADVACKCHGVSGSCSLKTCWLQLADFRKVGDALKEKYDGASRVLYGNRGSN
RASRAELLRLEPEDPAHKPPSPHDLVYIDPSPDYCVRNESTGSLGTQGRLCNKTSEGMDGCELMCCGRGYD
QFKTVQTERCHCKFHWCCYVKCKKCTEIVDQFVCK

Wnt3a_haXWnt8:

MAPLGYFLLLCSLKQALGSYPIWWSLAVGPQYSSLGSQPILCASIPGLVPKQLRFCRNYVEIMPSVAEGIK
IGIQECQHQFRGRRWNCTTVHDSLAIFGPVLDKATRESAFVHAIASAGVAFAVTRSCAEGTAAICGCSSRH
QGSPGKGWKWGGCSEDIEFGGMVSREFADARENRPDARSAMNRHNNEAGRQAIASHMHLKCKCHGLSGSCE
VKTCWWSQPDFRAIGDFLKDKYDQALKLEMDKRKMPSTNSVNSRRAIADAFSSVAGSELIYYEASPNFCEP
NPETGSFGTRDRTCNVSSHGIDGCDLLCCGRGHNARAERRREKCRCVFHWCCYVSCQECTRVYDVHTCK
